# Bone Organoids: A Novel Tool for Modeling and Managing Skeletal Disorders in Diabetes

**DOI:** 10.1002/advs.202518788

**Published:** 2026-03-25

**Authors:** Shuangzhe Lin, Karthikeyan Narayanan, Deepak Vashishth

**Affiliations:** ^1^ RPI – Icahn School of Medicine at Mount Sinai Center for Engineering and Precision Medicine New York NY USA; ^2^ Department of Biomedical Engineering Rensselaer Polytechnic Institute (RPI) Troy NY USA; ^3^ Shirley Ann Jackson Ph.D. Center of Biotechnology and Interdisciplinary Studies Troy New York USA

**Keywords:** bone organoids, diabetes‐associated bone fragility

## Abstract

Decades of research have revealed the profound impact of diabetes on skeletal health, yet the biological basis of diabetic bone fragility remains incompletely understood, and no existing in vitro or animal model has faithfully recapitulated these disease phenotypes. Organoid technologies are emerging as powerful tools for investigating human biology, modeling disease, and developing novel therapies. While organoids for many tissue types have been developed and continue to advance rapidly due to a growing regulatory shift toward human‐relevant in vitro models, bone organoids remain underrepresented, with only a few models described to date. Bone organoids can be generated using cell‐based self‐assembly or scaffold‐based guided approaches, each capable of reconstructing key features of native bone, including its multicellular composition, coordinated remodeling processes, and hierarchical extracellular matrix organization. In this review, we examine progress toward bone organoid development within the broader landscape of soft‐tissue organoid innovation and then use diabetes‐associated skeletal disorders as a representative case study to highlight the unmet needs and illustrate how next‐generation bone organoids could advance the modeling and management of these complex diseases.

## Graphical abstract

This review synthesizes advances in bone organoid engineering and evaluates their potential to model diabetic bone fragility. Current in vitro and animal models do not fully recapitulate diabetes‐associated skeletal phenotypes. Cell‐based and scaffold‐based bone organoids are discussed as complementary approaches to reconstruct multicellular bone composition, coordinated remodeling, and hierarchical extracellular matrix organization in the context of diabetes‐related skeletal disorders.

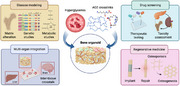

## 1 Introduction

Diabetes is a chronic disease in which the body produces insufficient insulin (a hormone produced by the pancreas) or responds abnormally to it, leading to elevated blood glucose levels [1]. In addition to well‐established complications affecting the heart, brain, nerves, eyes, and kidneys, increased skeletal fragility in bone is emerging as another clinically significant consequence of diabetes, particularly in elderly patients, who already face heightened risks for falls and fractures [2, 3, 4, 5]. Bone integrity depends on the tightly regulated process of remodeling coordinated by bone‐resorbing osteoclasts and bone‐forming osteoblasts [6]; disruption of this balance frequently leads to metabolic bone disorders, such as diabetes‐induced bone fragility [2, 3] and postmenopausal osteoporosis [7]. In diabetes, bone fragility is consistently associated with low bone turnover [2, 8], yet the underlying mechanisms remain poorly understood [9]. Moreover, osteocytes–the most abundant cells in bone and functionally central regulators of remodeling–are increasingly implicated in diabetic skeletal dysfunction [2, 3] but remain difficult to study due to their deep entrapment within the mineralized matrix and rapid loss of phenotype in traditional two‐dimensional (2D) culture [10].

Owing to the rigid and mineralized nature of bone, its inner space is anatomically difficult to access and often requires invasive techniques for in vivo examination [11]. Although vertebrate animal models are widely used to study human skeletal development and disease, they often fail to accurately recapitulate the structural complexity of human bone, highlighting the need for more physiologically relevant human models [12]. In the meantime, advances in three‐dimensional (3D) culture methods, including spheroid‐based models [13, 14, 15], biomimetic scaffolds [16, 17, 18] and microfluidic organ‐on‐a‐chip (OoC) platforms [19, 20, 21, 22], have enabled more predictive in vitro models and laid the groundwork for bioengineered organoids derived from human tissue and stem cells.

Organoids are 3D in vitro structures typically derived from pluripotent stem cells (PSCs), neonatal, or adult stem cells (ASCs) that self‐organize into assemblies recapitulating key aspects of organ structure and function [23]. Recent legislative initiatives [24, 25], together with ongoing efforts to reduce reliance on animal testing through OoC and organoid technologies [26], are driving a paradigm shift in disease modeling and therapeutic development. These advances also enable the culture of patient‐derived tissues in biomimetic 3D environments, supporting personalized approaches to drug testing. In bone research, however, a fully characterized organoid model that integrates all major bone cell types within a mineralized matrix has yet to be achieved [27]. Moreover, disease‐specific bone organoids tailored to model complex metabolic disorders, such as diabetic bone disease, remain scarce, underscoring the challenges of reconstructing mineralized, multicellular, and metabolically perturbed skeletal environments in vitro.

In light of these technical hurdles, this review synthesizes the fundamental biology that defines the requirements for bone organoid models and examines current engineering strategies, including cell‐based and scaffold‐based approaches. We then focus on skeletal disorders in diabetes as a representative case to illustrate how next‐generation bone organoids could advance disease modeling, therapeutic testing, and personalized medicine, thereby highlighting the broader potential of these systems to address longstanding challenges in bone research.

## 2 Bone Biology: Basis for Bone Organoids

**FIGURE 1:**
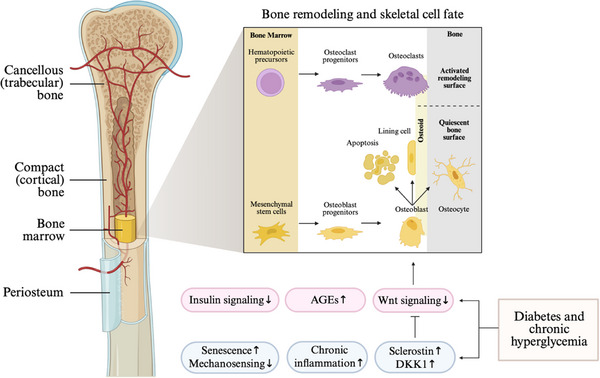
Bone biology and pathophysiology in diabetes. The complex anatomy of bone presents unique research interests and challenges in understanding its biology and associated diseases. Bone remodeling, a precisely coordinated process, involves the interplay of osteoblasts, osteoclasts, and osteocytes. Diabetes disrupts these cellular activities by inducing inflammation, producing advanced glycation end products (AGEs), disrupting signaling pathways, and ultimately compromising bone quality and leading to fragility.

In early multicellular life, structural support was minimal, and nutrient exchange occurred largely through absorption. As organisms grew more complex, specialized organs emerged along with the need for coordinated communication and protection. These evolutionary pressures drove the development of skeletal systems. In vertebrates, the skeleton performs vital functions, including providing support, assisting movement, producing blood cells, and regulating homeostasis of calcium and hormones. Anatomically, bone has an outer surface (i.e., periosteum) enclosing the inner space that is organized as compact and cancellous bone (Figure 1). Compact (cortical) bone is dense and composed of osteons surrounded by interstitial bone, whereas cancellous (trabecular) bone is highly porous and composed of a network of trabeculae [28]. Osteons, as the main structural units of compact bone, are organized in concentric layers of lamellae around a central cavity known as the Haversian canal, which is parallel to the long axis of the bone [29]. Osteons help impede fracture propagation by obstructing crack passage as it traverses the many osteons [30]. At the sub‐microstructural level, collagen triple helices assemble into fibrils that are mineralized with calcium hydroxyapatite (HAP) and stabilized by crosslinks, forming a composite matrix that provides bone with strength and flexibility [30].

In addition to these structural features optimized to meet mechanical demands, bone contains specialized cellular components–mainly osteoclasts, osteoblasts and osteocytes–that mediate skeletal growth, adaptation and repair. Bone is energetically expensive to build and maintain; remodeling requires coordinated resorption of existing mineralized matrix and deposition of new tissue. This tightly regulated process, known as bone remodeling, depends on the interplay between osteoclasts derived from hematopoietic precursors cells located at or near the bone surface [31] and osteoblasts derived from mesenchymal stem cells (MSCs) residing in the bone marrow [32]. These cells operate within temporary anatomical structures called basic multicellular units (BMUs), whose spatial and temporal organization ensures proper coupling between resorption and formation [33]. During remodeling, osteoclasts first resorb damaged or aged bone. Osteoblasts then repopulate the resorbed surface, synthesizing new matrix over a process that spans approximately 3 months. Upon completion, osteoblasts may become quiescent lining cells, undergo apoptosis, or become embedded within the newly formed osteons as osteocytes [33] (Figure 1).

Importantly, osteocytes account for 90%–95% of all bone cells and orchestrate skeletal physiology by forming an extensive cellular network within the mineralized matrix. Each osteocyte extends 40–100 elongated dendritic processes through narrow channels known as canaliculi, which measure approximately 250–300 nm in diameter [34, 35]. Through these processes, osteocytes form gap junction‐mediated connections with neighboring osteocytes, pericytes in blood capillaries, and surface‐resident bone cells such as bone lining cells, osteoclasts, and osteoblasts [35]. Osteocytes in trabecular bone typically exhibit a more rounded morphology, whereas those in cortical bone are elongated and aligned with the local lamellar matrix architecture [36].

Functionally, osteocytes are uniquely positioned to detect mechanical strain, matrix and mineral changes, and the formation of microcracks during daily activity. They respond to cracks by secreting chemotactic factors such as osteopontin (OPN) and recruiting MSCs to aid in the repair [37]. They can also respond to hormonal signals (e.g., parathyroid hormone (PTH) and estrogen) by modulating sclerostin (SOST) and activating Wnt/β‐catenin pathway for bone formation [38]. Osteoblasts integrate these signals from osteocytes to regulate osteoclastogenesis by producing macrophage colony‐stimulating factor (M‐CSF), osteoprotegerin (OPG), and receptor activator of NF‐κB ligand (RANKL) [38, 39, 40]. Although mechanisms and key coupling molecules, such as insulin‐like growth factors (IGFs) and transforming growth factor‐β (TGF‐β), have been proposed to mediate communication between osteoclasts and osteoblast‐lineage cells [41], a more precise understanding is still needed and could inform the development of effective therapeutic strategies [42].

Therefore, understanding the hierarchical structure, matrix composition, and physiological processes of bone is essential for both investigating skeletal diseases and guiding bone organoid design. In particular, the precise mechanisms by which bone cells sense, communicate, and respond within the 3D mineralized matrix remain difficult to study due to the inaccessibility of the osteocyte network in vivo [10]. These limitations underscore the need for advanced culture platforms capable of reconstructing bone's multicellular composition, spatial organization, and dynamic remodeling, motivating the development of bone organoids as physiologically relevant models for studying healthy and diseased skeletal function.

## 3 Bone Organoid Engineering

### 3.1 Historical Perspective

Over the past two decades, the broader field of organoid engineering has advanced rapidly, with numerous conceptual and technical innovations developed for soft‐tissue organoids now informing emerging efforts to construct bone organoids [43]. Organoids can be derived from either pluripotent stem cells (PSCs)–including embryonic stem cells (ESCs) and induced pluripotent stem cells (iPSCs)–or from tissue‐specific adult stem cells (ASCs). PSC‐derived organoids are generated by guiding cells through organ‐specific developmental trajectories using staged activation of lineage‐defining signaling pathways. In parallel, “outside‐in” cues from extracellular matrices are essential for maintaining polarity, spatial organization, and long‐term structural stability [44, 45]. ASCs isolated directly from postnatal or adult tissues provide an alternative route for generating organoids. These progenitors self‐organize into tissue‐specific structures when cultured in media supplemented with defined growth factor cocktails that maintain the stem cell niche. Intestinal organoids derived from LGR5^+^ ASCs were among the first examples [46, 47, 48], and refinements to these culture methods enabled ASC‐derived organoids from many soft tissues such as liver, colon, lung, stomach, pancreas, prostrate, ovaries and taste bud [49]. Importantly, PSC‐based systems tend to model early developmental or fetal stages, whereas ASC‐derived organoids more faithfully represent the adult tissue state.

### 3.2 Definition and Concept of Bone Organoids

Wang et al. have proposed distinguishing between pathological bone organoids, designed to model disease‐specific features for mechanistic studies and drug screening, and structural bone organoids, intended to recapitulate native bone architecture and functionality for repair and regeneration applications [50]. Building on this framework, we further categorize bone organoids into cell‐based and scaffold‐based systems, reflecting two major engineering routes for reconstructing key aspects of bone biology or modifying them to model skeletal pathologies.

Cell‐based (i.e., scaffold‐free) bone organoids rely on the intrinsic self‐organizing capacity of skeletal cells to form stable and complex structures without the use of external polymeric scaffolds or encapsulating hydrogels [51], making them particularly well‐suited for investigating developmental processes, mineralization pathways, and performing small‐molecule screening in order to identify compounds that modulate osteogenesis or pathological calcification [52]. In contrast, scaffold‐based organoids incorporate natural or synthetic biomaterials to guide cellular organization, enabling improved structural integrity and tunable mechanical properties [53]. Given the anatomical and functional heterogeneity of native bone–where cortical bone is dense and load‐bearing, and trabecular bone is porous and metabolically active–the required degree of mineralization and mechanical performance should be defined by the biological question being addressed. Accordingly, compared with cell‐based organoids, scaffold‐based organoids with enhanced mechanical strength and well‐ordered mineralization may be better suited for translational applications, such as implantation and repair of large segmental bone defects (LSBD) [54].

Beyond cortical and trabecular bone microarchitecture, more advanced organoids can recapitulate additional key functional features and anatomic niches of bone, including the bone marrow niche (endosteal/endosteum) [55], which provides a supportive milieu for hematopoietic stem cells; the periosteum, a fibrous and vascularized outer layer relevant for modeling fracture healing and callus formation [56]; and Haversian systems that support vascularization and nutrient exchange [57]. Such designs may further incorporate interactions with surrounding tissues to capture paracrine crosstalk within the skeletal microenvironment, as well as Schwann cell‐mediated innervation to better support osteogenesis and bone wound healing following implantation [58].

### 3.3 Cell‐Based Approaches

Engineering cell‐based bone organoids involves culturing cells, derived from healthy donors or patients with skeletal disorders, in 3D without exogenous solid supports (Table 1). This approach mitigates the challenges of seeding cells into porous scaffolds, where cell distribution is often inhomogeneous and initial density is low. The bottom‐up process used in scaffold‐free method depends on the inherent ability of the building blocks (e.g., spheroids, cell sheets) that can be combined and fuse into larger cohesive constructs [59]. With recent advances, 3D cell culture differentiation methods have been developed and used successfully to differentiate MSCs into functional bone forming cells [13, 14]. PSC aggregates (ESCs and iPSCs) cultured on non‐adherent surfaces can be induced to undergo spontaneous differentiation into MSC‐like cells, and PSC‐derived MSCs (iMSCs) can be obtained using either fetal bovine serum [60, 61, 62, 63, 64] or platelet lysate [65, 66]. The functional capabilities of iMSCs have been verified in various animal models such as limb ischemia [60, 64] or osteogenesis [62]. Most of the differentiation strategies utilize cellular signaling pathways involved in bone development, such as retinoic acid (RA), dexamethasone (DEX), bone morphogenetic proteins (BMPs) and Wnt [67]. This MSCs‐derived differentiation is a critical process, reflecting the developmental origin of mineralized tissues from the mesoderm lineage in vertebrates [68], and providing a practical approach to create bone organoids.

**TABLE 1:** Examples of cell‐based bone organoids.

Methods	Cell type(s)	Purposes	Refs.
Cell spheroid formation	Rat MSCs	To demonstrate enhanced osteoregenerative potential both in vitro and in vivo	[79]
	Human MSCs	To generate a rapidly mineralized cell model system for studying mineralization mechanisms and small molecule perturbations	[52]
Cell spheroid fusion	Differentiated rat osteoblasts	To assemble a 3D bone macrotissue by guided fusion of spheroids, allowing them to grow and mature without a scaffold	[74]
Cell sheet technique	BMSC‐derived ECs and undifferentiated BMSCs	To fabricate vascularized bone tissue and evaluates its potential to repair critical‐sized calvarial defects in rats	[77]
Patient‐derived cells	Patient‐derived primary cells or iPSCs	To understand the underlying genetic variations in diabetes and model patient‐specific disease pathophysiology	[161]
Genetically engineered cells	Human PSCs	To correct diabetes‐related point mutations or generate KOs of key genes in the pathological pathways	[163]
Chemical stimulation	MSCs or ESCs derived osteoblasts and pre‐osteoblasts	To reproduce diabetic phenotypes by exposing cells to hyperglycemia and/or oxidative conditions	[164, 165, 166, 167]

The principle of cellular spheroid formation is to prevent cells from adhering on the substrates, thereby promoting cellular self‐assembly [69]. Human MSCs have been cultured into spheroids that rapidly mineralize upon supplementation with calcium and phosphate ions into the culture medium [52]. Unlike a hydrogel matrix that tends to absorb mineralization factors [52], MSC spheroids formed in microwell arrays with low‐binding surface modification (Pluronic F108 coating) minimize this sequestration. Importantly, MSC donor variability has been found to affect spheroid mineralization; therefore, human MSC spheroid models offer a precision medicine approach to study small molecule perturbations and underlying biological mechanisms using patient‐specific specimens [52].

**FIGURE 2:**
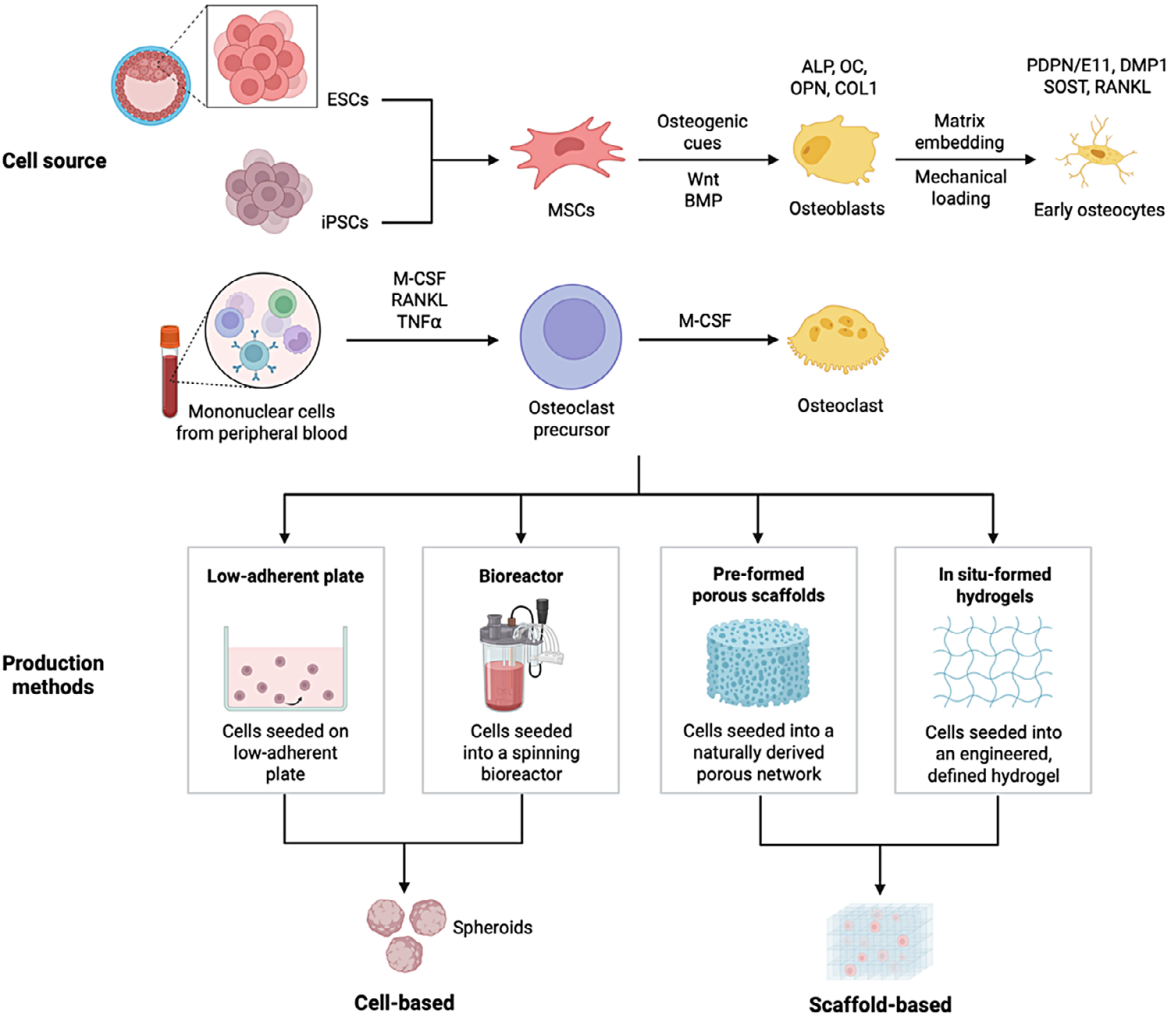
Engineering of bone organoids. Several biofabrication strategies have been developed to generate 3D bone organoids. These systems broadly fall into two categories: cell‐based, scaffold‐free models and scaffold‐based models incorporating biomaterials derived from natural or synthetic sources. Cell‐based spheroids self‐assemble under biochemical osteogenic induction in low‐adherent plates [200], whereas reported bioreactor systems apply cyclic loading or vibrational stimulation to promote osteogenic maturation [100]. In scaffold‐based approaches, scaffold stiffness and architectural features influence bone cell differentiation [201, 202], while engineered hydrogel matrices enable controlled composition and tunable porosity [203]. ESCs, embryonic stem cells; iPSCs, induced pluripotent stem cells; MSCs, mesenchymal stem cells; ECs, endothelial cells.

Moreover, incorporating systems that recapitulate bone resorption is equally important for investigating related therapeutic strategies [70]. Osteoclastic precursors can be obtained from bone marrow or peripheral blood mononuclear cells (PBMCs). Human monocytes derived from cord blood [71] or cell lines such as U‐937 [70] can be differentiated into multinucleated osteoclasts upon stimulation with M‐CSF and RANKL (Figure 2). Last, osteoclasts may also be isolated from native bone tissue, such as human bone biopsies from giant cell tumors [72]. Because bone formation and resorption are tightly coupled through signaling pathways such as the RANK‐RANKL–where RANK receptors expressed on osteoclasts respond to soluble RANKL produced by osteocytes [73]–integrating multiple bone cell types is essential to model coordinated bone remodeling units.

Recent engineering efforts have demonstrated spatial manipulation of bone cells to promote spheroid fusion; however, these systems have typically relied on a single cell type [74]. For example, scaffold‐free 3D bone macrotissues (>2.5 mm in size with mineral formation) have been generated through guided fusion of differentiated rat osteoblast spheroids using a removable 3D‐printed pillar array [74]. Although necrotic cores were observed, molecular assessment of the spheroids revealed osteogenic maturation, evidenced by osteocalcin (OC) expression (late differentiation marker expressed by mature osteoblasts and early osteocytes), downregulation of Runx2 (transcription factor of early osteoblast differentiation), and upregulated alkaline phosphatase (ALP) expression (marker for mineralization of the extracellular matrix). Moving forward, incorporating multiple bone cell populations through similar engineering approaches will be critical to more faithfully recapitulate BMUs during bone remodeling, particularly given that bone remodeling and osteocyte communication are disrupted in many skeletal diseases [75].

Furthermore, functional microvascular networks are crucial for the development of advanced bone organoids as they are known to mediate crosstalk between bone cell precursors [76]. One notable approach toward vascularized bone constructs uses cell sheet technology [77], another scaffold‐free method in which cultured cells naturally form dense intercellular connections and are harvested as intact sheets containing both cells and their secreted extracellular matrix (ECM). These sheets can be detached using thermo‐responsive surfaces, polyvinylidene di‐fluoride membranes, or ethylene diamine tetraacetic acid (EDTA) treatment [78]. For example, endothelial cells (ECs) derived from bone marrow MSCs (BMSCs) were seeded onto an undifferentiated BMSC sheet, forming lumen‐like structures after 3 days of co‐culture [77]. High‐density BMSCs were further differentiated to generate an osteogenic sheet, and the two layers were stacked to create a vascularized, scaffold‐free bone construct [77]. By retaining endogenous ECM, cell sheets more closely mimic the physiological bone niche, promote cell‐cell interactions, and enable direct transplantation with reduced immune rejection.

In addition, mineralization is another key feature targeted for characterization [52, 74, 77, 79] and can be induced by supplements such as ascorbic acid, hydrocortisone or DEX, β‐glycerophosphate, and ionic calcium and phosphate. Mineralization of bone organoids can be verified through Alizarin Red (AR) staining or fluorescent labeling with dyes like OsteoImage [80]. Scanning electron microscopy (SEM) with energy‐dispersive X‐ray spectroscopy (EDS), Fourier‐transform infrared spectroscopy (FTIR), Raman spectroscopy, and focused ion beam‐SEM (FIB‐SEM) imaging have also been used to elucidate the nature of mineralization and to identify the chemical composition of the deposited minerals [52].

Although cell‐based organoids show promise as organotypic 3D tissue surrogates in vitro, their use in disease modeling and therapeutic screening still requires substantial optimization and validation. Considerable efforts have focused on optimizing oxygen delivery to maintain appropriate stem cell fate and support ECM synthesis and deposition [81, 82]. Mechanical stimulation is equally critical, as tensile, compressive, and shear forces regulate ECM composition and overall tissue function. Together, these parameters can be leveraged to enhance matrix production and mineralization in developing bone organoids [83].

### 3.4 Scaffold‐Based Approaches

Scaffold‐based organoid engineering aims to develop elegant 3D tissue analogues using natural or synthetic polymeric structures that support initial attachment and proliferation of cells while promoting *de novo* formation of functional 3D tissue [59] (Figure 2). Traditional strategies employ cytocompatible, biodegradable and mechanically stable scaffolds with interconnected pores for efficient transport and exchange of oxygen, nutrients and metabolites [84]. Since cellular behaviors and functions are found to be influenced by the biochemistry and the nano‐ to micro‐scale surface topographies of the substrate [85], various biomaterials have been designed to optimize bone cell activities and provide favorable binding sites for attachment, proliferation, and differentiation [86].

One of the most notable natural biomaterials are derived from the ECM, the noncellular component of native tissues composed of a diverse array of proteins, including fibrillar proteins (e.g., collagens, elastin) that provide mechanical support; glycoproteins (e.g., fibronectin, laminins) that mediate cell‐ECM interactions; proteoglycans (e.g., perlecan, syndecan); noncollagenous proteins (e.g., OC, OPN [87]); and signaling factors such as TGF‐β and vascular endothelial growth factor (VEGF) that regulate neighboring cell activities. These ECM‐derived biomaterials used for engineering bone organoids include Matrigel and demineralized bone matrix (DBM). Matrigel, a basement membrane matrix derived from murine tumors, offers a collagen‐rich, biocompatible environment that can be supplemented with growth factors to support stem cell adhesion, proliferation, and lineage‐specific differentiation [88], whereas DBM can preserve the hierarchical collagen architecture of native bone [89].

Synthetic polymers offer the advantages of tunable mechanical properties, process control, and improved reproducibility [90]. Their stiffness spans several orders of magnitude, enabling further control of mechanical and biochemical properties for 3D bone cell culture. For example, polylactic acid (PLA) exhibits high mechanical strength (tensile modulus: 0.35–3.5 GPa) and is degradable via hydrolysis inside the human body. Polyglycolic acid (PGA; tensile modulus: 6–7 GPa) and its co‐polymer with PLA (PLGA) are both biodegradable and support cell migration, proliferation, and bone ingrowth. Other widely used synthetic materials include polycaprolactone (PCL; tensile modulus: 0.2–0.4 GPa), and highly water‐soluble polymers such as polyethylene glycol (PEG; tensile modulus: 0.12–0.266 GPa) and clickable polyvinyl alcohol (PVA) [91, 92]. Additionally, advanced hydrogel systems for bone organoid engineering, such as viscoelastic alginate‐collagen interpenetrating network (IPN) hydrogel [93]; photoresponsive hydrogels degradable by two‐photon, near‐infrared laser irradiation (700–900 nm) [94, 95]; and matrices formed by photopolymerization‐induced phase separation (PIPS) [96], represent promising tools for reconstructing physiologically relevant, interconnected 3D bone cell networks in vitro.

Scaffold‐based bone organoids can be further distinguished by how cells are spatially introduced and organized within the construct, most commonly through top seeding onto pre‐formed porous scaffolds or 3D embedding within hydrogels. Top‐seeding relies on scaffolds with large pores (∼100–600 µm) [97] that accommodate larger range of cell sizes and facilitate cell migration and vascularization throughout the scaffold; however, the cell‐surface interface is often effectively 2D, which can limit the formation of fully interconnected 3D cellular networks [18]. In contrast, 3D embedding strategies encapsulate cells within a surrounding matrix that provides biochemical and mechanical cues during differentiation. Conventional hydrogels used for 3D embedding typically exhibit nanoscale pore sizes (<100 nm) and limited permeability [98], so cell spreading often depends on matrix degradation. Accordingly, metalloproteinase (MMP)‐sensitive hydrogels [18, 99] have been developed to facilitate cell‐mediated remodeling. Together, these distinct organizational modes influence cell‐matrix interactions, nutrient transport, and the progression of osteogenic maturation, and therefore represent important design considerations in bone organoid engineering.

Having considered the selection of appropriate cell sources, biomaterials, and modes of cell introduction, it is now instructive to examine pioneering scaffold‐based approaches for engineering functional bone model systems (Table 2). These efforts require careful coordination of cellular differentiation with the deposition and mineralization of an organic matrix to achieve hierarchical bone‐like architecture. One of the first examples is the woven‐bone organoid reported by Hofmann et al., in which primary human BMSCs were seeded onto porous silk fibroin (SF) scaffolds and subjected to spinner‐flask mechanical stimulation to promote osteogenic differentiation [16]. Multiscale imaging showed that cells became embedded within a mineralized ECM and extended long processes (>10 µm), with contacts reported between 1 and 7 neighboring cells [16]. Osteocyte‐lineage progression was interpreted in this system based on the expression of early osteocyte markers, such as dentin matrix protein 1 (DMP1) and podoplanin (PDPN or E11 [16]. While SOST expression was also reported in this system, evidence for fully mature osteocyte phenotypes remains limited and requires further validation [16].

**TABLE 2:** Examples of scaffold‐based bone‐related organoids.

Cell type(s)	Scaffold materials	Purposes	Refs.
Primary human BMSCs	SF	To form an early stage mineralized bone model containing differentiated osteoblasts and osteocytes	[16]
Primary human osteoblasts and human dermal microvascular ECs	SF	To form microvasculature in bone scaffold material and promote in vivo angiogenesis upon implantation	[204]
Human MSCs	GO/alginate/gelatin	To demonstrate functional osteocyte differentiation and network formation under cyclic mechanical loading	[17]
Human MSCs	PEG hydrogel containing dextran, HA, fibronectin‐derived RGD peptide and MMP‐sensitive di‐cysteine peptide	To generate and in situ‐forming microporous scaffold that enables ultrafast bone cell spreading and network formation	[18]
Human and rat MSCs	SF	To generate cortical‐bone‐like lamellae and promote vascularization for repair of LSBD	[54]
Human BMSCs and human HUVECs	GelMA and PEG diacrylamide	To reconstruct osteonal structures including the concentric lamellae and Haversian canals	[110]
Primary rabbit BMSCs and endothelial progenitor cells derived from BMMs	PCL and bioink composed of gelatin, fibrinogen, HA, and glycerol	To reconstruct osteonal structures with vertically central medullary canals, Haversian canals, and transverse Volkmann canals	[205]
Primary rat BMSCs and HUVECs	GelMA and the zwitterionic monomer 2‐(methacryloyloxy) ethyl choline phosphate	To replicate the cellular spatial organization of native osteons through a dual‐ring hydrogel scaffold to promote synergistic angiogenesis and osteogenesis	[206]
Primary murine osteoblasts and BMMs	DBM	To reproduce bone tissue complexity and cellular processes in the trabecular bone niche	[89]
Human iPSCs	Hydrogels composed of 60% collagen and 40% Matrigel	To generate a bone marrow organoid capable of supporting the long‐term growth of primary cells from patients with myeloid and lymphoid blood cancers	[112]
Human iPSCs	Mixed hydrogel containing collagen I, collagen IV and Matrigel	To generate a 3D bone marrow model containing differentiated MSCs, fibroblasts, ECs and hematopoietic cells by a series of cytokine cocktails	[113]
Human iPSCs and primary human HSPCs	HAP scaffold	To generate a macro‐scale organotypic model of the bone marrow endosteal niche with integrated vascularization and a mineralized matrix	[55]

BMSCs: bone marrow mesenchymal stem cells; SF: silk fibroin; ECs: endothelial cells; MSCs: mesenchymal stem cells; GO: graphene oxide; PEG: polyethylene glycol; HA: hyaluronic acid; RGD: arginine‐glycine‐aspartic acid; MMP: metalloproteinase; LSBD: large segmental bone defects; HUVECs: human umbilical vein endothelial cells; GelMA: gelatin methacryloyl; BMMs: bone marrow mononuclear cells; PCL: polycaprolactone; DBM: demineralized bone matrix; iPSCs: induced pluripotent stem cells; HSPCs: hematopoietic stem and progenitor cells; HAP: hydroxyapatite.

Moreover, Zhang et al. engineered a bioprinted osteocyte organoid using a graphene oxide (GO)/alginate/gelatin composite bioink and demonstrated the critical role of mechanical loading in osteocyte lineage progression [17]. Cyclic loading (5 min per day, five times per week) was required to promote osteogenic maturation and the development of osteocyte‐like morphology. Notably, initiating mechanical stimulation on day 1 rather than delaying until day 21 significantly increased organoid mineral density and stiffness and enhanced the transition from osteoblasts to osteocyte‐like states. Early loading resulted in greater cell spreading and significantly longer dendrite processes (120.06 ± 22.18 µm) compared with late loading (55.92 ± 14.30 µm) or no loading (80.56 ± 12.83 µm), along with a higher percentage of cell connections. While PDPN expression was detected across all conditions, mechanical loading upregulated osteocyte‐associated markers DMP1 [17]. Furthermore, SOST was detected, however, its presence alone does not establish the formation of fully mature osteocytes, and additional investigations are required to demonstrate a functional osteocyte network operating within the engineered organoid [17]. This work highlights the importance and need of establishing early mechanobiological cues to promote osteocyte‐lineage differentiation and matrix mineralization, which remain critical for establishing a functional engineered bone organoid. Consistent with this concept, more recent studies have incorporated bioreactors to deliver cyclic stress or vibrational stimuli to bone organoids to better approximate the mechanical environment of native bone, although such systems remain at an early stage of development and require further optimization [100].

Another innovative strategy has focused on refining synthetic scaffolds to overcome the restricted mass transport and limited cell spreading imposed by the nanoscale porosity of conventional hydrogels. Zauchner et al. addressed this challenge by developing a matrix MMP‐degradable PEG hydrogel that undergoes in situ micropore formation through thiol‐Michael‐addition crosslinking in the presence of hyaluronic acid (HA) and dextran [18]. This process yields an interconnected microporous architecture (5–20 µm) that can be tuned by adjusting dextran concentration and molecular weight, thereby enabling improved permeability through the matrix. As a result, cells exhibited ultrafast spreading within 1.5 h and formed robust 3D networks within 24 h, underscoring how pore‐scale engineering can directly facilitate physiologically relevant bone cell connectivity.

In parallel, high‐resolution 3D bioprinting technologies have emerged as another powerful tool for engineering bone organoids with precise spatial control, high cell viability, and the ability to construct microscale structures [88]. For example, Lewis and colleagues pioneered a sacrificial ink approach that, through multi‐nozzle or coaxial bioprinting, enables the fabrication of hollow, perfused internal architectures following ink removal, and allows for the direct formation of vascular networks within organoids [101]. Other technologies, including ultrasonic bioprinting that leverages the deep penetration capability of acoustic waves and rapid acoustothermal‐induced free radical polymerization to achieve non‐contact solidification of bioinks into predesigned geometries [102], as well as microfluidic‐assisted 3D bioprinting that enables precise manipulation of microdroplets, molecules, or cells within bioinks, further support the generation of structured, cell‐laden constructs for bone tissue regeneration [103].

Recent advances in volumetric bioprinting (VBP) also enable rapid fabrication of geometrically intricate, centimeter‐scale hydrogel constructs within seconds via tomographic light projection, where dynamic light patterns simultaneously solidify an entire photoresponsive polymer volume [104]. This approach has been used to generate anatomically accurate trabecular bone models with embedded angiogenic sprouts, as well as complex meniscal grafts [105]. Gehlen et al. demonstrated that printing parameters (e.g., resin concentration and laser dose) affect cell behavior and tissue formation [106]. They identified an optimal gelatin methacryloyl (GelMA) bioresin formulation with lithium phenyl(2,4,6‐trimethylbenzoyl)phosphinate (LAP) as the photoinitiator (i.e., 5% GelMA and 0.05% LAP) and combined it with 3D endothelial co‐culture to generate a heterocellular, bone‐like construct with enhanced osteogenic differentiation and functionality [106]. Although increasing polymer concentration in gelatin‐based photoresins has been found to improve printability, it also results in a stiffer printed microenvironment that restricts 3D cell growth. This limitation was addressed by Qiu et al., who developed a synthetic PVA‐based resin incorporating thiolated PEG to ensure initial print fidelity, followed by thermal removal of gelatin to yield a stress‐relaxing PVA matrix [107]. This approach supported rapid cell spreading (within 2 h), osteogenic differentiation of embedded human MSCs, formation of 3D bone cell networks, and matrix mineralization [107].

Also, print fidelity depends on precise control of light distribution within the bioresin, which is challenged by scattering from embedded cells and mineral particles [108]. Using a polymer‐induced liquid‐phase precursor (PILP) strategy, researchers achieved in situ mineralization of GelMA hydrogels containing high cell densities (up to 3 million cells/ml) [109]. Poly‐aspartic acid stabilized amorphous mineral precursors, reduced light scattering, and enabled precise volumetric printing of thick, mineralizing constructs. Further tuning the refractive index (RI) of the bioresin produced uniformly mineralized, cell‐viable bone‐like matrices. Encapsulated human MSCs differentiated toward an early osteocytic phenotype within 28 days, demonstrating that VBP supports both high‐density cell embedding and controlled mineralized matrix formation.

Given the heterogeneity of bone, in vitro models must account for the distinct structural and functional characteristics of different bone tissue types. One study reported a cortical bone‐like construct generated using freeze‐casting of SF, and seeded with high densities of MSC‐derived osteoblasts [54]. These organoids promoted neovascularization by inducing macrophage polarization from the pro‐inflammatory M1 to the pro‐regenerative M2 phenotype, and enhanced bone regeneration during the LSBD repair [54]. In addition, scaffolds emulating hierarchical osteon structures have been engineered using advanced biofabrication techniques, including electrospinning, micropatterning, and laser‐directed microfabrication [110]. BMSCs cultured within these systems adopted osteocyte‐like morphologies, with cell bodies localized on microislands and dendritic extensions projecting into microchannels. Subsequent matrix rolling strategies recreated concentric lamellae and enabled investigation of intercellular communication across adjacent lamellae, while human umbilical vein endothelial cells (HUVECs)/Matrigel injection simulated Haversian canal formation within the osteon core [110]. Additional efforts to recapitulate osteon architecture are summarized in Table 2. These systems successfully reproduce many structural features of osteons; however, they do not yet recapitulate the full biological functionality required to be considered as cortical bone organoids.

For trabecular bone, its organoids have been modeled via co‐culturing primary murine osteoblasts and bone marrow mononuclear cells (BMMs) on thin slices of DBM [89]. Here, the osteoblasts were directed to deposit structural, mineralized bone tissue and subsequently acquire the resting‐state bone lining cell phenotype, similar to that seen in in vivo osteoid bone [89]. The bone lining cells were able to revert to an active state, becoming osteoblasts, and regaining osteogenic activity upon scratching the surface of the bone lining cells. They then shifted their secretory profile in response to chemical stimulation, inducing BMMs to differentiate into osteoclasts [89]. Therefore, such bone organoids reproduce the repeated bone remodeling processes in the trabecular bone niche.

Last, another major function of bone is hematopoiesis, which is maintained within the bone marrow niche that provides vascular, osteogenic, and hematopoietic progenitors and gives rise to diverse blood and immune cell lineages [111]. Because these cell populations are critical for connective tissue regeneration and vascularization, incorporating bone marrow‐derived elements is an important step toward building more physiologically complete bone organoids. Earlier studies [112, 113] demonstrated the generation of multilineage bone marrow organoids from human iPSCs by using staged cytokine cocktails and Matrigel to direct differentiation. These systems produced vascularized architectures containing MSCs, fibroblasts, endothelial and hematopoietic cells, partially recapitulating aspects of native bone marrow and enabling in vitro studies of bone biology, remodeling and pathology. However, these organoids remain micrometer scale, limiting the physiological relevance of their vascular structures, and they rely on Matrigel, which introduces mouse‐derived components [114].

More recently, human iPSCs combined with a porous HAP scaffold have been used to generate a macro‐scale vascularized osteoblastic organoid that models the trabecular architecture of the native bone marrow endosteal niche close to the bone surface [55]. This model generates long‐lasting vascular networks with pericyte coverage, neural fibers, and a mineralized osteogenic matrix. Importantly, it supports in vitro hematopoiesis and preserves the multilineage repopulating capacity of human hematopoietic stem and progenitor cells (HSPCs) in vivo. By addressing limitations in scale, vascular physiology, and matrix composition, this model represents a significant advance toward a uniform, durable, and reproducible human bone organoid with improved utility for disease modeling and drug screening.

## 4 Skeletal Disorders in Diabetes: A Case for Bone Organoid Applications

### 4.1 How Diabetes Alters Bone

Type 1 diabetes (T1D) is characterized by an autoimmune destruction of pancreatic β cells, leading to deficient insulin production and typically manifesting during childhood [115]. In contrast, type 2 diabetes (T2D) arises when the body cannot properly use insulin due to insulin resistance, and it is highly prevalent among older adults [116]. In T1D, bone mineral density (BMD) and bone strength are reduced, resulting in an elevated lifetime fracture risk [117]; this effect is especially pronounced at the hip and is thought to arise partly from failure to achieve optimal peak bone mass during growth due to the lack of insulin's anabolic effects [118]. In contrast, T2D presents a paradoxical phenotype in which fracture risk is increased despite normal or even elevated BMD [119] as measured by dual‐energy X‐ray absorptiometry (DXA) [120]. Moreover, the most widely used fracture risk assessment tool (FRAX), evaluating prior fragility fracture, parental history of hip fracture, smoking, glucocorticoid use, rheumatoid arthritis and alcohol consumption, has underestimated fracture risk in T2D patients [121]. Thus, factors beyond BMD, including bone mineralization, collagen characteristics, and bone tissue mechanical properties, likely contribute to skeletal fragility in T2D. However, the complexity and heterogeneity of these alterations make it challenging to fully elucidate their roles in human patients [122].

Cellular activities in bone such as bone remodeling and osteocyte communication are also profoundly influenced by the metabolic and hormonal disturbances in diabetic patients, where low bone turnover has been identified as a common feature [123]. Key discoveries have been made on the Wnt signaling that leads to bone formation, along with its inhibitors SOST and dickkopf‐related protein 1 (DKK1), both of which suppress osteoblast differentiation and are consistently found to be high in the serum collected from T1D and T2D patients [124, 125, 126, 127] (Figure 1). However, results on whether osteoclastic bone resorption is suppressed or increased in diabetes are not consistent and the process remains poorly understood despite the known impact of high‐glucose‐induced proinflammatory state on osteoclast‐mediated pathogenesis [128]. Moreover, elevated glucose significantly increases SOST and its mRNA in osteocytes [129]. A recent study using biochemical markers has shown that in T1D rodents, low bone turnover causes increased apoptosis of osteocytes, disruption of osteocyte canaliculi, and accumulation of microdamage, causing bone fragility [130]. In addition, RANKL production from the osteocytes is reduced, suggesting the communication between apoptotic osteocytes and nearby viable cells is interrupted. Hence, osteoclast recruitment is suppressed and osteoblast differentiation is impaired [130].

In addition, hyperglycemia leads to an increased production of AGEs, which are a diverse group of chemical compounds resulting from a nonenzymatic glycation of amino acids such as lysine and arginine with the electrophilic carbonyls of reducing sugars, known as the Maillard reaction [131]. Collagen in bone is particularly susceptible to formation of AGEs such as pentosidine and N‐carboxymethyllysine (CML) [87, 132, 133]. The accumulation of AGEs in collagen can alter its crosslinking and function, stiffening its structures, making it more resistant to tissue remodeling, and leading to a brittle bone that is vulnerable to fracture upon mechanical loading [134]. Furthermore, AGEs can bind to their receptor (RAGE) and decrease bone cell functions, thus worsening the reduced tissue turnover and causing more AGE accumulation in diabetes [135, 136]. It has been shown that interrupting the AGEs/RAGE pathway ameliorates diabetic bone damage in mice, suggesting AGEs as a potential therapeutic target for managing and treating diabetes‐related bone diseases [137].

Concurrently, aging that often accompanies and is accelerated by diabetes [138] is characterized by well‐established molecular and cellular hallmarks–including genomic instability, epigenetic drift, mitochondrial dysfunction, and cellular senescence. These interrelated processes disrupt tissue homeostasis and impair regenerative capacity across organ systems [139]. Within the skeletal niche, aging profoundly alters the bone marrow microenvironment, shifting the balance of cues required for osteogenesis and bone remodeling. Importantly, many of these aging‐related alterations–low bone turnover, impaired osteoblast function, and increased marrow adiposity–overlap with pathogenic mechanisms observed in diabetic bone [2, 4], highlighting shared vulnerabilities in the aging and diabetic skeleton.

A central feature of skeletal aging is the “bone‐fat imbalance,” in which bone marrow MSCs increasingly favor adipogenic over osteogenic differentiation. This shift is driven in part by lineage‐specific transcriptional changes: PPARγ and C/EBP⍺ promote adipogenic differentiation, whereas Runx2 and Osterix (Osx/Sp7) drive osteogenesis. In aged BMSCs, elevated PPARγ expression enhances adipogenic differentiation while suppressing osteogenesis [140], whereas Runx2 and Osx are significantly reduced [141, 142]. Metabolic alterations–including increased lipid oxidation and accumulation of lipotoxic species such as palmitate–further promote oxidative stress (OS), upregulate PPARγ, and suppress pro‐osteogenic Wnt signaling [143, 144]. Biophysical factors within the aging bone marrow niche also play a key role: the mechanical stiffness of the extracellular matrix has been shown to direct MSC fate, with softer matrices favoring adipogenesis and stiffer matrices promoting osteogenesis [145]. Together, these biochemical and biomechanical changes reinforce adipogenic dominance, compromise bone formation, and increase skeletal fragility in the aged bone. Notably, similar patterns of impaired osteogenesis, lipotoxic stress, mitochondrial dysfunction, and altered mechanotransduction are also hallmarks of diabetic bone, suggesting that aging and diabetes may act synergistically to exacerbate bone fragility through convergent pathways [146].

### 4.2 Limitations of Current Models to Study Diabetic Bone Diseases

As bone is one of the hardest tissues in the human body, where mineral obscures the complex inner hierarchical structure, its inaccessibility hinders progress in understanding bone biology and disease. Due to the limitations of current imaging technology, it is impossible for the bone matrix to be imaged while in a live patient. Currently, traditional laboratory research methods rely on animal experiments and 2D cell culture of mammalian cells (Table 3). Animal models can reflect the complexity of the human body; however, their physiology still diverges from the human state and fails to capture the heterogeneity of the human population [147]. At the same time, 2D cell culture offers simplistic interactions upon exposure to drugs or other molecules, but far away from representing the natural physiological microenvironments [148].

**TABLE 3:** Conventional strategies for studying diabetic bone diseases.

	Methods	Advantages	Disadvantages	Applications	Refs.
Animal					
	Chemical ablation of the β cells	Quick and relatively inexpensive; Easily reproducible	Toxic at other organs of the body; Can revert to normal glucose levels after a few weeks	Achieves the deficiency of insulin production in T1D; Chemically induced by toxins such as STZ or alloxan	[150]
	Spontaneous autoimmune T1D model	Shares parallel genes with T1D humans	Unpredictable disease onset; More expensive to maintain	Helps understand the underlying genetics and mechanisms of T1D; NOD mice and BB rats	[207]
	Genetically engineered obese T2D model	Matches the pathophysiology of human patients; Represents the human condition more realistically from polygenic model	Fails to capture the heterogeneity of the human population by monogenic model	Evaluates novel treatments of T2D and diabetic complications; Monogenic (e.g., Lep^ob/ob^ mice and Lepr^db/db^ mice) and polygenic (e.g., KK mice and OLETF rat)	[208]
	Induction by high fat feeding	Reflects obesity induced by environmental manipulation	Heterogeneous response to the feeding diet even within the same inbred	Results in obesity, insulin resistance, altered glucose tolerance and T2D in C57BL/6J mice	[152]
Fixed bone section	Histological and cytochemical examination	Preserves microarchitecture and morphology of bone tissue; Reduces the number of animals used	Static images offer only a limited insight into the cellular activities	Identifies specific structural changes associated with diseased conditions; Uses specific stains to assess osteoblast and osteoclast activity	[209]
Bone explant	Extraction from animal fetuses or young pups	Maintains the complete cellular composition in its native environment; Preserves cell‐cell and cell‐matrix interactions	Lack of vasculature; Requires similar number of experimental animals	Investigates the effects of mechanical loading; Establishes ex vivo bone defect models to evaluate biomaterial implants	[210]
2D cell culture	Monolayer of bone cells	Offers simplistic interactions upon exposure to drugs or other molecules	Deviates from the 3D natural bone microenvironments; Often leads to erroneous observations and results	Mimics T2D by plating primary human osteoblasts on culture plastic and exposed to high concentrations of insulin and/or glucose	[156, 157, 158, 211]

T1D: type 1 diabetes; STZ: streptozotocin; NOD: non‐obese diabetic; BB: BioBreeding; T2D: type 2 diabetes; Lep: leptin; ob/ob: obese; Lepr: leptin receptor; db/db: diabetic; KK: Kasukabe strain; OLETF: Otsuka Long‐Evans Tokushima Fatty.

Numerous animal models have been developed to mimic the skeletal characteristic of human diabetes. One of the most widely used models of T1D is the spontaneous diabetic Wistar rat, commonly known as the BioBreeding (BB) rat. First described in 1974 and more extensively characterized in 1978 by Nakhooda and colleagues, this strain shares parallel genes with T1D humans, including the major histocompatibility complex (MHC) class II that confers susceptibility to T1D [149]. The deficiency in insulin production can also be achieved by chemical ablation of the β cells through an exposure to toxins including streptozotocin (STZ) or alloxan. They provide simple and relatively cheap models, but the chemicals can be toxic to other organs of the body and can affect P450 isozymes in various places [150].

T2D animal models tend to include features of insulin resistance and/or β cell failure, and many T2D animals are obese. This obesity can be induced by genetic manipulation or through high fat feeding. Genetically obese models for T2D include monogenic mice such as Lep^ob/ob^ (deficient in leptin that induces satiety) and Lepr^db/db^ (deficient in leptin receptor), and polygenic rodents such as KK mice and Otsuka Long‐Evans Tokushima Fat (OLETF) rat. Despite the current use of monogenic models for T2D research, obesity in humans is rarely caused by a monogenic mutation. However, a major concern of using polygenic models is the lack of wild‐type controls [151]. High fat feeding (58% energy by fat) to C57BL/6J mice is a well‐characterized model that results in obesity, insulin resistance, altered glucose tolerance and therefore T2D [152], although heterogeneous responses to high fat feeding have been reported within the inbred C57BL/6J strain [153].

While animal models are pivotal for investigating diabetes and its complications, several drugs that showed effective in animal models have failed to show efficacy in human clinical trials [154]. In addition, cortical bone differs structurally between small mammals and humans [155], raising the question of whether mouse models are useful for identifying therapeutic approaches to strengthen cortical bone, a critical determinant of bone's fracture propensity. 2D cell culture has long been used to grow bone cells as monolayers or in co‐culture systems. For example, in 2012, an in vitro 2D model mimicking the onset of T2D was described, where primary human osteoblasts were plated on culture plastic and exposed to high concentrations of insulin and/or glucose [156]. Similarly, human primary osteoclasts plated on the surface of bovine cortical bone or ivory dentin slices have been used to monitor the inhibition of bone‐resorbing activities by novel drugs [157]. Co‐cultures of bone cells with other cell types have led to several important advances; for example, outgrowth ECs (OECs) isolated from human peripheral blood formed organized, lumen‐containing pre‐vascular networks when directly co‐cultured with primary human osteoblasts on fibronectin‐coated glass coverslips under osteogenic differentiation conditions [158].

Although 2D cultures have revealed many important aspects of bone biology and disease mechanisms, they fail to reproduce the comprehensive bone microenvironment and rely on conventional plastic substrates that lack the organic (collagen‐rich) and inorganic (HAP‐based) matrix components essential for bone cell differentiation, signaling, and function, often leading to erroneous observations and results. These limitations have motivated the development of bioengineered 3D culture systems designed to better capture the spatial complexity and coordinated remodeling processes of bone. Such organotypic models also reduce reliance on costly and imperfect animal models and offer greater clinical relevance when built from human‐derived tissues and cells.

### 4.3 How Bone Organoids Could Advance Diabetes Research and Management

Clinical evaluation of diabetic bone disease currently relies on imaging‐based assessments of bone mass and quality using tools such as DXA, high‐resolution computed tomography (HRCT), and magnetic resonance imaging (MRI). In contrast, bone organoids allow a broader range of characterization techniques that are impractical in vivo. For example, micro‐CT scanning or micro‐MRI can be applied to organoids for 3D reconstruction and quantitative assessment of morphology, density, and microarchitecture [159]. Additionally, histological and immunohistochemical analyses–including hematoxylin and eosin (H&E), Masson's trichrome, and antibody‐based staining–can be directly performed on organoid sections to evaluate matrix organization, mineralization, and cellular composition with high spatial resolution.

**FIGURE 3:**
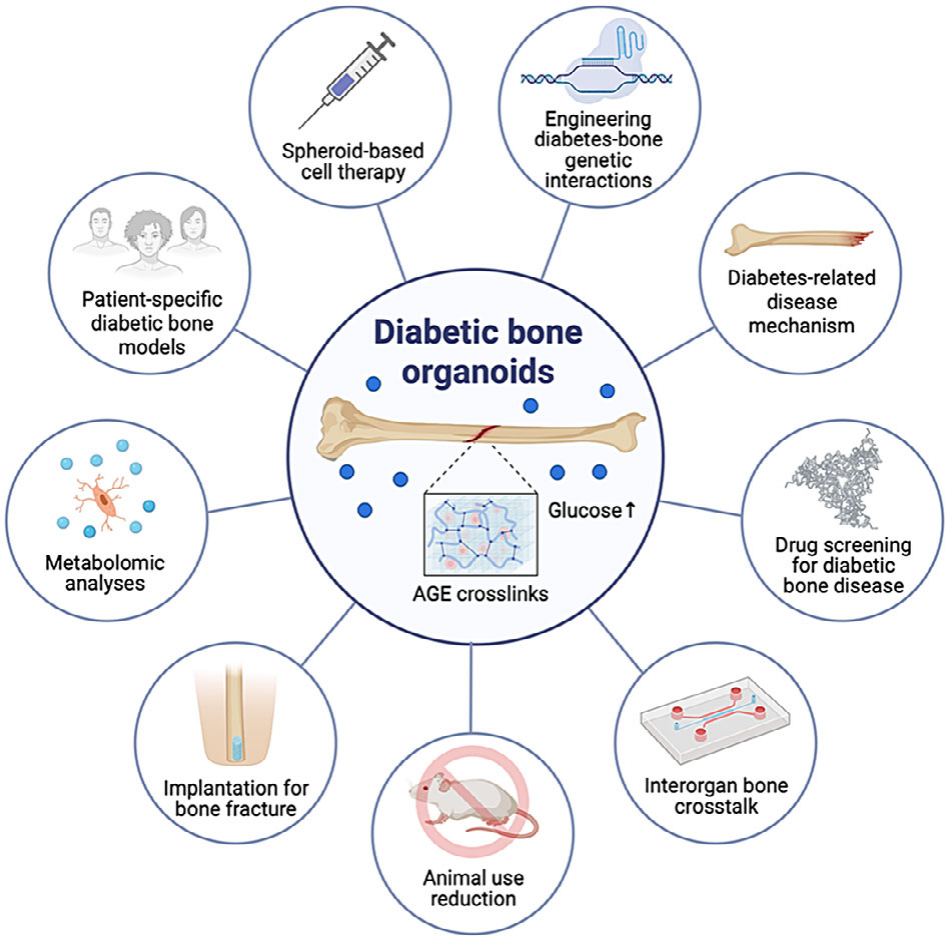
Applications of diabetic bone organoids. Bone organoids for diabetes, though still rare, will provide a human‐relevant platform for modeling hyperglycemia‐induced matrix alterations, including advanced glycation end‐product (AGE) crosslinking, and for dissecting diabetes‐bone interactions. These systems will enable investigation of disease mechanisms, metabolic profiling, and patient‐specific modeling, while supporting drug screening for the treatment of diabetic bone fragility. Integration with microfluidic chips facilitates studies of pre‐diabetic effects on specific organs and inter‐tissue crosstalk. They may also be adapted for implantation strategies and regenerative applications. Collectively, such models offer scalable in vitro systems that can reduce animal use and help bridge the translational gap between conventional cell culture and clinical studies.

Clinically, diabetic bone is characterized by suppressed bone turnover. A recent systematic review and meta‐analysis comprising 66 studies (62 cross‐sectional, 3 randomized controlled trials, 1 longitudinal study involving 16–890 patients, predominantly with T2D) confirmed that individuals with diabetes exhibit significantly lower levels of bone turnover markers (mean difference [95% CI]: CTX −0.10 ng/mL [−0.12 to −0.08], PINP −10.80 ng/mL [−12.83 to −8.77]) [160]. Bone organoids generated from patient‐derived primary cells or iPSCs hold promise for recapitulating these hallmarks, providing a controllable system to study the cellular and molecular basis of low‐turnover bone disease in diabetes (Figure 3).

#### 4.3.1 Fundamental Research

Bone organoids derived from either patient‐specific primary cells or iPSCs offer a unique platform for dissecting diabetes‐associated skeletal defects at a mechanistic level. Unlike animal models, these organoids retain patient‐specific genomic and epigenomic signatures–including noncoding variants and regulatory elements implicated in diabetes risk–allowing investigation of 3D chromatin hubs and enhancer‐promoter interactions relevant to diabetic bone fragility [161]. Genetic editing technologies such as CRISPR‐Cas9 further enable precise correction or introduction of diabetes‐associated mutations, as well as targeted knockouts of genes central to bone cell dysfunction [162, 163].

Modeling diabetic pathology in organoids can be achieved through controlled hyperglycemic exposure, which simulates chronic metabolic stress. Extended high‐glucose culture (>7 days) reduces proliferation and osteogenic differentiation in MSC‐ or ESC‐derived osteoblasts [164, 165, 166, 167], while also inducing inflammation, VEGF upregulation, and reactive oxygen species (ROS) accumulation–hallmarks of diabetic bone disease [168, 169]. Long‐term transcriptomic profiling revealed coordinated downregulation of ECM synthesis, lysosomal activity, and cell viability pathways under persistent hyperglycemia, alongside increased OS [170]. Furthermore, the ability to engineer AGE accumulation within bone organoids represents another significant advantage. Because AGEs drive many diabetic complications [171], in vitro glycation, controlled by sugar type, temperature, concentration, and additives, together with enhanced AGE‐RAGE interactions [172], provides a tunable system to replicate early and late stages of diabetic bone matrix deterioration [132, 173]. Together, genetically engineered, patient‐derived, and metabolically stressed bone organoids enable a level of mechanistic fidelity and experimental flexibility unattainable with traditional models.

#### 4.3.2 Understanding the Crosstalk Between Bone and Other Organs

Bone diseases frequently emerge from disruptions in mineral deposition and calcium homeostasis, reflecting broader disturbances in hormonal and metabolic feedback loops. These insights have reshaped our understanding of bone as an active endocrine organ embedded within systemic metabolic networks. For example, osteoblasts express high levels of the insulin receptor (InsR), a tyrosine kinase that promotes osteoblast proliferation, collagen synthesis, and OC production [174]. Mice lacking InsR showed reduced bone volume, lower osteoblast and osteoclast activity [175], underscoring the critical role of insulin signaling in skeletal homeostasis. Reciprocally, undercarboxylated OC–released during bone resorption–promotes pancreatic β‐cell proliferation, insulin release [176], and glucagon‐like peptide‐1 (GLP‐1) secretion [177], establishing a bidirectional pancreas‐bone axis that links bone remodeling to systemic energy metabolism.

These findings underscore the need for OoC microfluidic platform that integrate bone with other tissues to recapitulate multiple‐organ physiology in vitro [19]. Recent advances–including 3D pancreatic islet perfusion chips capable of sustaining long‐term culture under physiologically relevant flow [20] and coupled pancreas‐liver systems enabling real‐time analysis of glucose‐responsive insulin secretion and hepatic glucose uptake [21, 22]–demonstrate the feasibility of such integration for studying early diabetic dysfunction. Extending these approaches to skeletal tissues will expand the toolkit for investigating diabetic complications at the organ level [178]. Such platforms could elucidate how chronic hyperglycemia, impaired insulin signaling, or altered OC bioactivity contribute to diabetic osteopathy. Microfluidic bone‐on‐chip systems already permit controlled osteoblast‐osteoclast‐osteocyte interactions, mechanical loading studies, and osteochondral interface modeling [179, 180], and could further enable interrogation of molecular crosstalk within the pancreas‐bone axis, including insulin resistance in osteoblasts, altered bone turnover, and disrupted hormone signaling.

#### 4.3.3 Drug Screening and Drug Toxicity Assessment

Traditional drug screening for bone diseases suffers from major limitations: 2D cultures fail to reproduce multicellular bone microenvironments, while animal models do not always predict human responses. Bone organoids offer a promising alternative, particularly in light of recent FDA support for reducing reliance on animal testing [24, 25, 26]. For example, intestinal organoids have been used to screen over 2000 compounds for inhibitors of ion transport [181] and liver cancer organoids have identified novel ERK inhibitor (SCH772984) as potential therapeutics [182]. Such examples demonstrate the promise of organoid platforms in enabling human‐relevant preclinical testing.

Drug‐induced adverse effects remain a major barrier to therapeutic development, underscoring the need for improved tools that can model drug responses in human tissues. For instance, glucocorticoid‐induced glucose intolerance–linked in part to reductions in OC [183]–illustrates how endocrine interactions between bone and metabolic organs can mediate systemic side effects. Organoids offer a route to detect such off‐target effects earlier. Cardiac organoids, for example, have been used to evaluate the cardiotoxicity of doxorubicin, revealing apoptosis and release of myocardial injury biomarkers [184]. However, current toxicity pipelines rarely assess skeletal toxicity, even though many commonly used drugs (e.g., warfarin, cyclosporine, chemotherapeutics) adversely affect bone metabolism. Radiological approaches can detect overt structural damage, but earlier changes in bone matrix production or cellular activity often remain unseen. A reliable bone organoid platform could fill this gap by enabling early detection of drug‐induced disruptions in bone turnover and matrix quality.

#### 4.3.4 Precision Medicine

Incorporating patient‐specific genetics into toxicity testing enhances drug therapeutic precision. Personalized organoids can identify individuals at heightened risk for bone toxicity or altered drug responses [185], similar to how patient‐derived tumor organoids are used to guide individualized chemotherapy [186, 187, 188, 189], and similar approaches could transform the management of primary bone tumors, metastatic bone diseases, osteoporosis, and diabetes‐associated bone fragility. Applying this precision framework to bone disease could significantly reduce adverse outcomes and improve therapeutic efficacy.

Patient‐derived organoids (PDOs) have rapidly emerged as promising tools for modeling human disease heterogeneity, enabling researchers to capture patient‐specific genetic, epigenetic, and phenotypic signatures in vitro. PDO technology was initially established in cancer and epithelial tissue [48, 182, 188] and has expanded to musculoskeletal research [16, 89]. In bone research, PDOs enable the study of patient‐specific differences in osteogenesis, bone remodeling, etc., that are lacking in traditional PSC or ASC derived organoid models. For example, Kim et al. developed PDOs for a rare bone disorder (fibrous dysplasia) and showed feasibility of faithful in vitro replication of the disease, which includes metabolic profiles and non‐aligned isotropic patterns of ECM [190]. Because PDOs preserve patient‐specific genetic risk factors–including those linked to diabetes, bone fragility, or poor fracture healing–they enable mechanistic studies that cannot be accomplished using animal models or generalized cell lines. PDOs not only offer a powerful platform for personalized medicine but also facilitate evaluation of inter‐individual variability in drug response. When combined with microfluidic organ‐on‐chip platforms, PDO‐integrated bone organoids can further enable multi‐organ crosstalk studies (e.g., pancreas‐bone axis). Thus, PDO‐enhanced bone organoids represent a transformative step toward precision modeling of diabetic bone disease and diabetic‐induced diseases such as cardiomyopathy, nephropathy, retinopathy, etc.

Ongoing technological advances in this area are accelerating, for example, bioprinting and artificial intelligence (AI) enhance structural fidelity, culture control, and analytical throughput, while biological tools such as gene editing expand cellular complexity and support precise disease modeling. 3D bioprinting provides spatial control over the arrangement of cells and biomaterials, allowing reconstruction of anatomically inspired features of healthy and diseased bone tissue [191, 192], including porous trabecular‐like structures and vascular channels. AI tools, including machine learning and deep learning, are increasingly applied to optimize induction parameters, automate culture monitoring, and enable high‐throughput, high‐precision analysis [193]. Gene editing technologies, most notably CRISPR/Cas9, offer programmable manipulation of lineage specification and pathogenic mutations, facilitating the creation of bone organoid models with well‐defined genetic backgrounds [194]. Together, these advances position patient‐specific bone organoids as a promising platform for precision diagnostics, therapeutic testing, and the development of personalized treatment strategies in skeletal medicine.

## 5 Challenges and Perspective

The emergence of bone organoid technologies marks a transformative step toward modeling skeletal complications associated with endocrine disorders such as diabetes. By recreating human‐relevant physiology, architecture, and disease phenotypes in 3D, bone organoids provide advantages over traditional cell cultures and animal models, including the ability to capture both biochemical and biophysical cues that regulate bone cell function and to more faithfully emulate physiological and pathological states. PDOs further enable investigation of inter‐individual variability in diabetes‐associated bone fragility and offer a platform for personalized therapeutic testing.

Recent advances in 3D bioprinting, gene editing, high‐resolution microscopy, multi‐omics profiling, and AI‐driven design will accelerate the development of increasingly sophisticated bone organoid systems. However, most current platforms still lack essential features of native bone, including adequate vascularization, appropriate mechanical loading, and full cellular heterogeneity. The lack of vascular structures restricts organoid size, compromises oxygen and nutrient transport, and limits both differentiation and long‐term viability. Likewise, conventional static culture conditions in suspension or hydrogels fail to deliver the dynamic mechanical cues required for physiological osteogenic maturation. Overall, current systems largely reproduce early bone formation processes such as woven bone or osteogenic differentiation, whereas the generation of fully functional cortical bone organoids with mature osteonal architecture and appropriate mechanical properties remains a challenge [195].

Another important area for investigation is the interaction between the microbiome and bone, as growing evidence links gut microbiome dysbiosis to metabolic bone disorders [196]. In this context, gut‐derived hormones, including GLP‐1, PTH, and glucose‐dependent insulinotropic polypeptide (GIP), together with shifts in microbial composition characterized by a reduction in beneficial bacterial taxa and an expansion of potentially pathogenic genera such as *Enterococcus*, play crucial roles in the pathogenesis of diabetic bone disease [197, 198]. While current bone organoids do not account for microbiome‐derived signals, future organoid and OoC platforms may enable investigation of this axis through controlled integration of microbial metabolites or multi‐organ coupling [199].

Additional challenges persist in standardizing culture systems, improving reproducibility, and achieving scalability and cost‐effectiveness for disease modeling and high‐throughput drug screening. Variability in cytokine combinations used for stem cell maintenance further complicates protocol harmonization, and there is still no consensus on selecting material systems tailored to specific research goals. Moreover, many of the newest technologies have been demonstrated primarily in murine cell models, and their applicability for primary human cell‐derived systems has yet to be fully validated.

In summary, the continued integration of multidisciplinary innovations in stem cell biology, biomaterials engineering, microfluidics, and computational modeling is moving bone niche reconstruction toward more precise and functional in vitro systems. Addressing the current limitations will be essential for establishing bone organoids as standardized tools for elucidating endocrine‐related bone pathology and accelerating the discovery of therapeutic interventions.

## Conflicts of Interest

The authors declare no conflict of interest.

## Data Availability

The authors have nothing to report.

## References

[1] B. S. Beydag‐Tasöz , S. Yennek , and A. Grapin‐Botton , “Towards a Better Understanding of Diabetes Mellitus Using Organoid Models,” Nature Reviews Endocrinology 19 (2023): 232–248. 10.1038/s41574-022-00797-x PMC9857923 36670309

[2] L. C. Hofbauer , B. Busse , R. Eastell , et al., “Bone Fragility in Diabetes: Novel Concepts and Clinical Implications,” The Lancet Diabetes & Endocrinology 10 (2022): 207–220. 35101185 10.1016/S2213-8587(21)00347-8

[3] N. Napoli , M. Chandran , D. D. Pierroz , B. Abrahamsen , A. V. Schwartz , and S. L. Ferrari , “Mechanisms of Diabetes Mellitus‐induced Bone Fragility,” Nature Reviews Endocrinology 13 (2017): 208–219. 10.1038/nrendo.2016.153 27658727

[4] M. R. Rubin , “Bone Cells and Bone Turnover in Diabetes Mellitus,” Current Osteoporosis Reports 13 (2015): 186–191. 25740570 10.1007/s11914-015-0265-0

[5] C. Hamann , S. Kirschner , K.‐P. Günther , and L. C. B. Hofbauer , “Bone, Sweet Bone—Osteoporotic Fractures in Diabetes Mellitus,” Nature Reviews Endocrinology 8 (2012): 297–305. 10.1038/nrendo.2011.233 22249517

[6] N. Kohli , S. Ho , S. J. Brown , et al., “Bone Remodelling In Vitro: Where Are We Headed?,” Bone 110 (2018): 38–46. 29355746 10.1016/j.bone.2018.01.015

[7] M. M. Weivoda , C. K. Chew , D. G. Monroe , et al., “Identification of Osteoclast‐Osteoblast Coupling Factors in Humans Reveals Links between Bone and Energy Metabolism,” Nature Communications 11 (2020): 87. 10.1038/s41467-019-14003-6 PMC6946812 31911667

[8] B. Lecka‐Czernik , “Diabetes, Bone and Glucose‐Lowering Agents: Basic Biology,” Diabetologia 60 (2017): 1163–1169. 28434032 10.1007/s00125-017-4269-4 PMC5487688

[9] V. V. Shanbhogue , D. M. Mitchell , C. J. Rosen , and M. L. Bouxsein , “Type 2 Diabetes and the Skeleton: New Insights into Sweet Bones,” The Lancet Diabetes & Endocrinology 4 (2016): 159–173. 26365605 10.1016/S2213-8587(15)00283-1

[10] N. Sawa , H. Fujimoto , Y. Sawa , and J. Yamashita , “Alternating Differentiation and Dedifferentiation Between Mature Osteoblasts and Osteocytes,” Scientific Reports 9 (2019): 13842. 31554848 10.1038/s41598-019-50236-7 PMC6761144

[11] P. M. Gilbert , S. Hofmann , H.‐H. Ng , H. Vankelecom , and J. M. Wells , “Organoids in Endocrine and Metabolic Research: Current and Emerging Applications,” Nature Reviews Endocrinology 1–7 (2024): 195–201, 10.1038/s41574-023-00933-1. 38182746

[12] A. I. Pearce , R. G. Richards , S. Milz , E. Schneider , and S. G. Pearce , “Animal Models for Implant Biomaterial Research in Bone: a Review,” European Cells and Materials 13 (2007): 1–10. 17334975 10.22203/ecm.v013a01

[13] A. P. D. N. de Barros , C. M. Takiya , L. R. Garzoni , et al., “Osteoblasts and Bone Marrow Mesenchymal Stromal Cells Control Hematopoietic Stem Cell Migration and Proliferation in 3D In Vitro Model,” PLoS ONE 5 (2010): 9093. 10.1371/journal.pone.0009093 PMC2816998 20161704

[14] J. E. Frith , B. Thomson , and P. G. Genever , “Dynamic Three‐Dimensional Culture Methods Enhance Mesenchymal Stem Cell Properties and Increase Therapeutic Potential,” Tissue Engineering Part C: Methods 16 (2010): 735–749. 19811095 10.1089/ten.TEC.2009.0432

[15] W. Wang , K. Itaka , S. Ohba , et al., “3D spheroid Culture System on Micropatterned Substrates for Improved Differentiation Efficiency of Multipotent Mesenchymal Stem Cells,” Biomaterials 30 (2009): 2705–2715. 19215979 10.1016/j.biomaterials.2009.01.030

[16] A. Akiva , J. Melke , S. Ansari , et al., “An Organoid for Woven Bone,” Advanced Functional Materials 31 (2021): 2010524.

[17] J. Zhang , J. Griesbach , M. Ganeyev , et al., “Long‐Term Mechanical Loading Is Required for the Formation of 3D Bioprinted Functional Osteocyte Bone Organoids,” Biofabrication 14 (2022): 035018. 10.1088/1758-5090/ac73b9 35617929

[18] D. Zauchner , M. Z. Müller , M. Horrer , et al., “Synthetic Biodegradable Microporous Hydrogels for In Vitro 3D Culture of Functional human Bone Cell Networks,” Nature Communications 15 (2024): 5027. 10.1038/s41467-024-49280-3 PMC11176307 38871693

[19] C. M. Leung , P. de Haan , K. Ronaldson‐Bouchard , et al., “A Guide to the Organ‐on‐a‐Chip,” Nature Reviews Methods Primers 2 (2022): 1–29.

[20] Y. Jun , J. Lee , S. Choi , et al., “In Vivo–Mimicking Microfluidic Perfusion Culture of Pancreatic Islet Spheroids,” Science Advances 5 (2019): aax4520. 10.1126/sciadv.aax4520 PMC6881167 31807701

[21] S. Bauer , C. Wennberg Huldt , K. P. Kanebratt , et al., “Functional Coupling of Human Pancreatic Islets and Liver Spheroids On‐a‐Chip: Towards a Novel human Ex Vivo Type 2 Diabetes Model,” Scientific Reports 7 (2017): 14620. 29097671 10.1038/s41598-017-14815-w PMC5668271

[22] R. Zandi Shafagh , S. Youhanna , J. Keulen , et al., “Bioengineered Pancreas–Liver Crosstalk in a Microfluidic Coculture Chip Identifies Human Metabolic Response Signatures in Prediabetic Hyperglycemia,” Advanced Science 9 (2022): 2203368. 36285680 10.1002/advs.202203368 PMC9731722

[23] M. Huch , J. A. Knoblich , M. P. Lutolf , and A. Martinez‐Arias , “The Hope and the Hype of Organoid Research,” Development 144 (2017): 938–941. 28292837 10.1242/dev.150201

[24] A. Mullard , “Is The Fda's Plan to Phase Out Animal Toxicity Testing Realistic?,” Nature Reviews Drug Discovery 24 (2025): 413–414. 10.1038/d41573-025-00087-x 40374990

[25] D. M. Stresser , A. K. Kopec , P. Hewitt , et al., “Towards In Vitro Models for Reducing or Replacing the Use of Animals in Drug Testing,” Nature Biomedical Engineering 8 (2024): 930–935. 10.1038/s41551-023-01154-7 38151640

[26] FDA pushes to replace animal testing , Nature Biotechnology 43, (2025): 655–655. 10.1038/s41587-025-02690-0 40380010

[27] M. G. Fois , M. van Griensven , S. Giselbrecht , P. Habibovic , R. K. Truckenmüller , and Z. N. Tahmasebi Birgani , “Mini‐Bones: Miniaturized Bone In Vitro Models,” Trends in Biotechnology 42 (2024): 910–928. 38493050 10.1016/j.tibtech.2024.01.004

[28] T. Hoenig , K. E. Ackerman , B. R. Beck , et al., “Bone Stress Injuries,” Nature Reviews Disease Primers 8 (2022): 1–20. 10.1038/s41572-022-00352-y PMC13227457 35484131

[29] N. Reznikov , M. Bilton , L. Lari , M. M. Stevens , and R. Kröger , “Fractal‐Like Hierarchical Organization of Bone Begins at the Nanoscale,” Science 360 (2018): aao2189. 10.1126/science.aao2189 PMC6037297 29724924

[30] E. Seeman and P. D. Delmas , “Bone Quality — The Material and Structural Basis of Bone Strength and Fragility,” New England Journal of Medicine 354 (2006): 2250–2261. 16723616 10.1056/NEJMra053077

[31] W. J. Boyle , W. S. Simonet , and D. L. Lacey , “Osteoclast Differentiation and Activation,” Nature 423 (2003): 337–342. 12748652 10.1038/nature01658

[32] M. Ponzetti and N. Rucci , “Osteoblast Differentiation and Signaling: Established Concepts and Emerging Topics,” International Journal of Molecular Sciences 22 (2021): 6651. 34206294 10.3390/ijms22136651 PMC8268587

[33] R. L. Jilka , “Biology of the Basic Multicellular Unit and the Pathophysiology of Osteoporosis,” Medical and Pediatric Oncology 41 (2003): 182–185. 12868116 10.1002/mpo.10334

[34] L. F. Bonewald , “Osteocytes as Dynamic Multifunctional Cells,” Annals of the New York Academy of Sciences 1116 (2007): 281–290. 17646259 10.1196/annals.1402.018

[35] I. Kalajzic , B. G. Matthews , E. Torreggiani , M. A. Harris , P. Divieti Pajevic , and S. E. Harris , “In Vitro and In Vivo Approaches to Study Osteocyte Biology,” Bone 54 (2013): 296–306. 23072918 10.1016/j.bone.2012.09.040 PMC3566324

[36] J. D. Currey , “The Many Adaptations of Bone,” Journal of Biomechanics 36 (2003): 1487–1495. 14499297 10.1016/s0021-9290(03)00124-6

[37] L. F. Raheja , D. C. Genetos , and C. E. Yellowley , “Hypoxic Osteocytes Recruit human MSCs through an OPN/CD44‐mediated Pathway,” Biochemical and Biophysical Research Communications 366 (2008): 1061–1066. 18155656 10.1016/j.bbrc.2007.12.076

[38] S. L. Dallas , M. Prideaux , and L. F. Bonewald , “The Osteocyte: An Endocrine Cell… and More,” Endocrine Reviews 34 (2013): 658–690. 23612223 10.1210/er.2012-1026 PMC3785641

[39] S. M. Khosla , “Minireview: the OPG/RANKL/RANK System,” Endocrinology 142 (2001): 5050–5055. 11713196 10.1210/endo.142.12.8536

[40] Y. L. Ma , R. L. Cain , D. L. Halladay , et al., “Catabolic Effects of Continuous Human PTH (1–38) in Vivo Is Associated with Sustained Stimulation of RANKL and Inhibition of Osteoprotegerin and Gene‐Associated Bone Formation,” Endocrinology 142 (2001): 4047–4054. 11517184 10.1210/endo.142.9.8356

[41] Y. Tang , X. Wu , W. Lei , et al., “TGF‐β1–induced Migration of Bone Mesenchymal Stem Cells Couples Bone Resorption with Formation,” Nature Medicine 15 (2009): 757–765. 10.1038/nm.1979 PMC2727637 19584867

[42] M. Wu , S. Wu , W. Chen , and Y.‐P. Li , “The Roles and Regulatory Mechanisms of TGF‐β and BMP Signaling in Bone and Cartilage Development, Homeostasis and Disease,” Cell Research 34 (2024): 101–123. 38267638 10.1038/s41422-023-00918-9 PMC10837209

[43] L. Bai , D. Zhou , G. Li , J. Liu , X. Chen , and J. Su , “Engineering Bone/Cartilage Organoids: Strategy, Progress, and Application,” Bone Research 12 (2024): 66. 39567500 10.1038/s41413-024-00376-y PMC11579019

[44] M. Huch and B.‐K. Koo , “Modeling Mouse and Human Development Using Organoid Cultures,” Development 142 (2015): 3113–3125. 26395140 10.1242/dev.118570

[45] M. A. Lancaster and J. A. Knoblich , “Organogenesis in a Dish: Modeling Development and Disease Using Organoid Technologies,” Science 345 (2014): 1247125. 25035496 10.1126/science.1247125

[46] N. Barker , J. H. van Es , J. Kuipers , et al., “Identification of Stem Cells in Small Intestine and Colon by Marker Gene Lgr5,” Nature 449 (2007): 1003–1007. 17934449 10.1038/nature06196

[47] N. Barker , M. Wetering , and H. Clevers , “The Intestinal Stem Cell,” Genes & Development 22 (2008): 1856–1864. 18628392 10.1101/gad.1674008 PMC2735277

[48] T. Sato , R. G. Vries , H. J. Snippert , et al., “Single Lgr5 Stem Cells Build Crypt‐villus Structures In Vitro without a Mesenchymal Niche,” Nature 459 (2009): 262–265. 19329995 10.1038/nature07935

[49] D. Dutta , I. Heo , and H. Clevers , “Disease Modeling in Stem Cell‐Derived 3D Organoid Systems,” Trends in Molecular Medicine 23 (2017): 393–410. 28341301 10.1016/j.molmed.2017.02.007

[50] J. Wang , X. Chen , R. Li , et al., “Standardization and Consensus in the Development and Application of Bone Organoids,” Theranostics 15 (2025): 682–706. 39744680 10.7150/thno.105840 PMC11671374

[51] X. Liu , Z. Zhou , Y. Zhang , H. Zhong , X. Cai , and R. Guan , “Recent Progress on the Organoids: Techniques, Advantages and Applications,” Biomedicine & Pharmacotherapy 185 (2025): 117942. 40043462 10.1016/j.biopha.2025.117942

[52] S. Vermeulen , K. Knoops , H. Duimel , et al., “An In Vitro Model System Based on Calcium‐ and Phosphate Ion‐Induced hMSC Spheroid Mineralization,” Materials Today Bio 23 (2023): 100844. 10.1016/j.mtbio.2023.100844 PMC10682137 38033367

[53] M. J. Kratochvil , A. J. Seymour , T. L. Li , S. P. Pasca , C. J. Kuo , and S. C. Heilshorn , “Engineered Materials for Organoid Systems,” Nature Reviews Materials 4 (2019): 606–622. 10.1038/s41578-019-0129-9 PMC7864216 33552558

[54] Y. Shuai , T. Yang , M. Zheng , et al., “Oriented Cortical‐Bone‐like Silk Protein Lamellae Effectively Repair Large Segmental Bone Defects in Pigs,” Advanced Materials 37 (2025): 2414543. 39871679 10.1002/adma.202414543 PMC11899506

[55] Q. Li , M. T. Nikolova , G. Zhang , et al., “Macro‐scale, Scaffold‐assisted Model of the human Bone Marrow Endosteal Niche Using hiPSC‐vascularized Osteoblastic Organoids,” Cell Stem Cell 32 (2025): 1941–1958. 41260216 10.1016/j.stem.2025.10.009

[56] G. Nilsson Hall , L. F. Mendes , C. Gklava , and L. Geris , “Developmentally Engineered Callus Organoid Bioassemblies Exhibit Predictive In Vivo Long Bone Healing,” Advanced Science 7 (2020): 1902295. 31993293 10.1002/advs.201902295 PMC6974953

[57] M. Zhang , R. Lin , X. Wang , et al., “3D Printing of Haversian Bone–Mimicking Scaffolds for Multicellular Delivery in Bone Regeneration,” Science Advances 6 (2020): aaz6725. 10.1126/sciadv.aaz6725 PMC7083611 32219170

[58] S. Song , J. Zhang , Y. Fang , et al., “Nerve–Bone Crosstalk Manipulates Bone Organoid Development and Bone Regeneration: A Review and Perspectives,” Organoid Research 1 (2025): 8294.

[59] A. Ovsianikov , A. Khademhosseini , and V. Mironov , “The Synergy of Scaffold‐Based and Scaffold‐Free Tissue Engineering Strategies,” Trends in Biotechnology 36 (2018): 348–357. 29475621 10.1016/j.tibtech.2018.01.005

[60] Q. Lian , Y. Zhang , J. Zhang , et al., “Functional Mesenchymal Stem Cells Derived From Human Induced Pluripotent Stem Cells Attenuate Limb Ischemia in Mice,” Circulation 121 (2010): 1113–1123. 20176987 10.1161/CIRCULATIONAHA.109.898312

[61] Y. Liu , A. J. Goldberg , J. E. Dennis , G. A. Gronowicz , and L. T. Kuhn , “One‐Step Derivation of Mesenchymal Stem Cell (MSC)‐Like Cells from Human Pluripotent Stem Cells on a Fibrillar Collagen Coating,” PLoS ONE 7 (2012): 33225. 10.1371/journal.pone.0033225 PMC3310052 22457746

[62] L. Zou , Y. Luo , M. Chen , et al., “A Simple Method for Deriving Functional MSCs and Applied for Osteogenesis in 3D Scaffolds,” Scientific Reports 3 (2013): 2243. 23873182 10.1038/srep02243 PMC3718204

[63] K. Hynes , D. Menicanin , K. Mrozik , S. Gronthos , and P. M. Bartold , “Generation of Functional Mesenchymal Stem Cells from Different Induced Pluripotent Stem Cell Lines,” Stem Cells and Development 23 (2014): 1084–1096. 24367908 10.1089/scd.2013.0111 PMC4015475

[64] G.‐W. Hu , Q. Li , X. Niu , et al., “Exosomes Secreted by human‐induced Pluripotent Stem Cell‐Derived Mesenchymal Stem Cells Attenuate Limb Ischemia by Promoting Angiogenesis in Mice,” Stem Cell Research & Therapy 6 (2015): 10. 26268554 10.1186/scrt546 PMC4533800

[65] J. Frobel , H. Hemeda , M. Lenz , et al., “Epigenetic Rejuvenation of Mesenchymal Stromal Cells Derived from Induced Pluripotent Stem Cells,” Stem Cell Reports 3 (2014): 414–422. 25241740 10.1016/j.stemcr.2014.07.003 PMC4266008

[66] C. Luzzani , G. Neiman , X. Garate , et al., “A Therapy‐grade Protocol for Differentiation of Pluripotent Stem Cells into Mesenchymal Stem Cells Using Platelet Lysate as Supplement,” Stem Cell Research & Therapy 6 (2015): 6. 25582222 10.1186/scrt540 PMC4417240

[67] S. Kawai , H. Yoshitomi , J. Sunaga , et al., “In Vitro Bone‐Like Nodules Generated From Patient‐Derived iPSCs Recapitulate Pathological Bone Phenotypes,” Nature Biomedical Engineering 3 (2019): 558–570. 10.1038/s41551-019-0410-7 31182836

[68] A. D. Berendsen and B. R. Olsen , “Bone Development,” Bone 80 (2015): 14–18. 26453494 10.1016/j.bone.2015.04.035 PMC4602167

[69] M. C. Decarli , R. Amaral , D. P. D. Santos , et al., “Cell Spheroids as a Versatile Research Platform: Formation Mechanisms, High Throughput Production, Characterization and Applications,” Biofabrication 13 (2021): 032002. 10.1088/1758-5090/abe6f2 33592595

[70] R. Detsch and A. R. Boccaccini , “The Role of Osteoclasts in Bone Tissue Engineering,” Journal of Tissue Engineering and Regenerative Medicine 9 (2015): 1133–1149. 24478169 10.1002/term.1851

[71] D. Fong , M. Bisson , G. Laberge , et al., “Bone Morphogenetic Protein‐9 Activates Smad and ERK Pathways and Supports Human Osteoclast Function and Survival In Vitro,” Cellular Signalling 25 (2013): 717–728. 23313128 10.1016/j.cellsig.2012.12.003

[72] F. Monchau , A. Lefèvre , M. Descamps , A. Belquin‐myrdycz , P. Laffargue , and H. F. Hildebrand , “In Vitro Studies of Human and Rat Osteoclast Activity on Hydroxyapatite, β‐Tricalcium Phosphate, Calcium Carbonate,” Biomolecular Engineering 19 (2002): 143–152. 12202175 10.1016/s1389-0344(02)00023-0

[73] G. Zhu , T. Zhang , M. Chen , et al., “Bone Physiological Microenvironment and Healing Mechanism: Basis for Future Bone‐Tissue Engineering Scaffolds,” Bioactive Materials 6 (2021): 4110–4140. 33997497 10.1016/j.bioactmat.2021.03.043 PMC8091181

[74] V. Prabhakaran , F. P. W. Melchels , L. M. Murray , and J. Z. Paxton , “Engineering Three‐Dimensional Bone Macro‐tissues by Guided Fusion of Cell Spheroids,” Frontiers in Endocrinology 14 (2023): 1308604. 38169965 10.3389/fendo.2023.1308604 PMC10758461

[75] T. Bellido , “Osteocyte‐Driven Bone Remodeling,” Calcified Tissue International 94 (2014): 25–34. 24002178 10.1007/s00223-013-9774-y PMC3947228

[76] J. Tuckermann and R. H. Adams , “The Endothelium–bone Axis in Development, Homeostasis and Bone and Joint Disease,” Nature Reviews Rheumatology 17 (2021): 608–620. 34480164 10.1038/s41584-021-00682-3 PMC7612477

[77] M. Xu , J. Li , X. Liu , et al., “Fabrication of Vascularized and Scaffold‐Free Bone Tissue Using Endothelial and Osteogenic Cells Differentiated From Bone Marrow Derived Mesenchymal Stem Cells,” Tissue and Cell 61 (2019): 21–29. 31759403 10.1016/j.tice.2019.08.003

[78] A. Alghuwainem , A. T. Alshareeda , and B. Alsowayan , “Scaffold‐Free 3‐D Cell Sheet Technique Bridges the Gap BETWeen 2‐D Cell Culture and Animal Models,” International Journal of Molecular Sciences 20 (2019): 4926. 31590325 10.3390/ijms20194926 PMC6801996

[79] Y. Yamaguchi , J. Ohno , A. Sato , H. Kido , and T. Fukushima , “Mesenchymal Stem Cell Spheroids Exhibit Enhanced In‐Vitro and In‐Vivo Osteoregenerative Potential,” BMC Biotechnology 14 (2014): 105. 25479895 10.1186/s12896-014-0105-9 PMC4299781

[80] F. Langenbach , K. Berr , C. Naujoks , et al., “Generation and Differentiation of Microtissues from Multipotent Precursor Cells for Use in Tissue Engineering,” Nature Protocols 6 (2011): 1726–1735. 22011655 10.1038/nprot.2011.394

[81] A. Mohyeldin , T. Garzón‐Muvdi , and A. Quiñones‐Hinojosa , “Oxygen in Stem Cell Biology: a Critical Component of the Stem Cell Niche,” Cell Stem Cell 7 (2010): 150–161. 20682444 10.1016/j.stem.2010.07.007

[82] P.‐J. Stiers , N. van Gastel , and G. Carmeliet , “Targeting the Hypoxic Response in Bone Tissue Engineering: A Balance Between Supply and Consumption to Improve Bone Regeneration,” Molecular and Cellular Endocrinology 432 (2016): 96–105. 26768117 10.1016/j.mce.2015.12.024

[83] M. A. Fernandez‐Yague , S. A. Abbah , L. McNamara , D. I. Zeugolis , A. Pandit , and M. J. Biggs , “Biomimetic Approaches in Bone Tissue Engineering: Integrating Biological and Physicomechanical Strategies,” Advanced Drug Delivery Reviews 84 (2015): 1–29. 25236302 10.1016/j.addr.2014.09.005

[84] S. J. Hollister , “Porous Scaffold Design for Tissue Engineering,” Nature Materials 4 (2005): 518–524. 16003400 10.1038/nmat1421

[85] N. Gjorevski , M. Nikolaev , T. E. Brown , et al., “Tissue Geometry Drives Deterministic Organoid Patterning,” Science 375 (2022): aaw9021. 10.1126/science.aaw9021 PMC9131435 34990240

[86] S. Wu , X. Liu , K. W. K. Yeung , C. Liu , and X. Yang , “Biomimetic Porous Scaffolds for Bone Tissue Engineering,” Materials Science and Engineering: R: Reports 80 (2014): 1–36.

[87] G. E. Sroga and D. Vashishth , “Effects of Bone Matrix Proteins on Fracture and Fragility in Osteoporosis,” Current Osteoporosis Reports 10 (2012): 141–150. 22535528 10.1007/s11914-012-0103-6 PMC3375270

[88] D. Gao , R. Li , J. Pan , et al., “3D Bioprinting Bone/Cartilage Organoids: Construction, Applications, and Challenges,” Journal of Orthopaedic Translation 55 (2025): 75–93. 40989070 10.1016/j.jot.2025.08.008 PMC12451290

[89] Y. Park , E. Cheong , J.‐G. Kwak , R. Carpenter , J.‐H. Shim , and J. Lee , “Trabecular Bone Organoid Model for Studying the Regulation of Localized Bone Remodeling,” Science Advances 7 (2021): abd6495. 10.1126/sciadv.abd6495 PMC7817107 33523925

[90] S. S. Manohar , C. Das , and V. Kakati , “Bone Tissue Engineering Scaffolds: Materials and Methods,” 3D Printing and Additive Manufacturing 11 (2024): 347–362. 38389691 10.1089/3dp.2022.0216 PMC10880649

[91] B. Wang , Y. Huang , Q. Cai , Z. Du , and X. Li , “Biomaterials for Diabetic Bone Repair: Influencing Mechanisms, Multi‐Aspect Progress and Future Prospects,” Composites Part B: Engineering 274 (2024): 111282.

[92] X.‐H. Qin , X. Wang , M. Rottmar , B. J. Nelson , and K. Maniura‐Weber , “Near‐Infrared Light‐Sensitive Polyvinyl Alcohol Hydrogel Photoresist for Spatiotemporal Control of Cell‐Instructive 3D Microenvironments,” Advanced Materials 30 (2018): 1705564. 10.1002/adma.201705564 29333748

[93] M. Bernero , D. Zauchner , R. Müller , and X.‐H. Qin , “Interpenetrating Network Hydrogels for Studying the Role of Matrix Viscoelasticity in 3D Osteocyte Morphogenesis,” Biomaterials Science 12 (2024): 919–932. 38231154 10.1039/d3bm01781h PMC10863643

[94] W. Qiu , C. Gehre , J. P. Nepomuceno , et al., “Coumarin‐Based Photodegradable Hydrogels Enable Two‐Photon Subtractive Biofabrication at 300 Mm s^−1^ ,” Angewandte Chemie International Edition 63 (2024): 202404599. 10.1002/anie.202404599 39023389

[95] C. Gehre , W. Qiu , P. Klaus Jäger , et al., “Guiding Bone Cell Network Formation in 3D via Photosensitized Two‐Photon Ablation,” Acta Biomaterialia 174 (2024): 141–152. 38061678 10.1016/j.actbio.2023.11.042

[96] M. Z. Müller , M. Bernero , C. Xie , et al., “Cell‐Guiding Microporous Hydrogels by Photopolymerization‐induced Phase Separation,” Nature Communications 16 (2025): 4923. 10.1038/s41467-025-60113-9 PMC12116776 40425560

[97] V. Karageorgiou and D. Kaplan , “Porosity of 3D Biomaterial Scaffolds and Osteogenesis,” Biomaterials 26 (2005): 5474–5491. 15860204 10.1016/j.biomaterials.2005.02.002

[98] K. J. De France , F. Xu , and T. Hoare , “Structured Macroporous Hydrogels: Progress, Challenges, and Opportunities,” Advanced Healthcare Materials 7 (2018): 1700927. 10.1002/adhm.201700927 29195022

[99] A. H. Aziz , R. L. Wilmoth , V. L. Ferguson , and S. J. Bryant , “IDG‐SW3 Osteocyte Differentiation and Bone Extracellular Matrix Deposition Are Enhanced in a 3D Matrix Metalloproteinase‐Sensitive Hydrogel,” ACS Applied Bio Materials 3 (2020): 1666–1680. 10.1021/acsabm.9b01227 PMC7384758 32719827

[100] X. Lou , Q. Zhou , Z. Dong , L. Bai , J. Su , and H. Yue , “Innovative Strategies for Bone Organoid: Synergistic Application and Exploration of Advanced Technologies,” Journal of Orthopaedic Translation 54 (2025): 180–198. 40836932 10.1016/j.jot.2025.07.010 PMC12362404

[101] P. P. Stankey , K. T. Kroll , A. J. Ainscough , et al., “Embedding Biomimetic Vascular Networks via Coaxial Sacrificial Writing Into Functional Tissue,” Advanced Materials 36 (2024): 2401528. 10.1002/adma.202401528 39092638

[102] X. Kuang , Q. Rong , S. Belal , et al., “Self‐Enhancing Sono‐Inks Enable Deep‐Penetration Acoustic Volumetric Printing,” Science 382 (2023): 1148–1155. 38060634 10.1126/science.adi1563 PMC11034850

[103] F. Serpe , L. Iafrate , M. Bastioli , et al., “Engineering a Microfluidic‐assisted 3D Bioprinting Approach for the Hierarchical Control Deposition and Compartmentalisation of Graded Bioinks,” Biofabrication 17 (2025): 045009. 10.1088/1758-5090/adf35b 40701169

[104] D. Loterie , P. Delrot , and C. Moser , “High‐resolution Tomographic Volumetric Additive Manufacturing,” Nature Communications 11 (2020): 852. 10.1038/s41467-020-14630-4 PMC7015946 32051409

[105] P. N. Bernal , P. Delrot , D. Loterie , et al., “Volumetric Bioprinting of Complex Living‐Tissue Constructs Within Seconds,” Advanced Materials 31 (2019): 1904209. 10.1002/adma.201904209 31423698

[106] J. Gehlen , W. Qiu , G. N. Schädli , R. Müller , and X.‐H. Qin , “Tomographic Volumetric Bioprinting of Heterocellular Bone‐Like Tissues in Seconds,” Acta Biomaterialia 156 (2023): 49–60. 35718102 10.1016/j.actbio.2022.06.020

[107] W. Qiu , J. Gehlen , M. Bernero , et al., “A Synthetic Dynamic Polyvinyl Alcohol Photoresin for Fast Volumetric Bioprinting of Functional Ultrasoft Hydrogel Constructs,” Advanced Functional Materials 33 (2023): 2214393.

[108] P. N. Bernal , M. Bouwmeester , J. M. Wolff , et al., “Volumetric Bioprinting of Organoids and Optically Tuned Hydrogels to Build Liver‐Like Metabolic Biofactories,” Advanced Materials 34 (2022): 2110054. 10.1002/adma.202110054 35166410

[109] B. W. M. Wildt , M. Bernero , D. Zauchner , R. Müller , and X.‐H. Qin , “Volumetric Bioprinting of Bone‐Like Mineralizing Hydrogel Constructs in the Presence of High Cell Densities and Mineral Precursors,” Biofabrication (2026), 10.1088/1758-5090/ae4ff7. 41806476

[110] X. Li , Y. Sun , S. Wang , C. Si , H. Li , and B. Chang , “A 3‐Dimensional Scaffolding System Recapitulates the Hierarchical Osteon Structure,” ACS Omega 9 (2024): 41368–41377. 39398190 10.1021/acsomega.4c04146 PMC11465375

[111] A. N. Tikhonova , I. Dolgalev , H. Hu , et al., “The Bone Marrow Microenvironment at Single‐cell Resolution,” Nature 569 (2019): 222–228. 30971824 10.1038/s41586-019-1104-8 PMC6607432

[112] A. O. Khan , A. Rodriguez‐Romera , J. S. Reyat , et al., “Human Bone Marrow Organoids for Disease Modeling, Discovery, and Validation of Therapeutic Targets in Hematologic Malignancies,” Cancer Discovery 13 (2023): 364–385. 36351055 10.1158/2159-8290.CD-22-0199 PMC9900323

[113] A.‐A. Olijnik , A. R. Romera , Z. C. Wong , et al., “Generating human Bone Marrow Organoids for Disease Modeling and Drug Discovery,” Nature Protocols (2024): 1–30, 10.1038/s41596-024-00971-7. 38532070

[114] S. Kim , S. Min , Y. S. Choi , et al., “Tissue Extracellular Matrix Hydrogels as Alternatives to Matrigel for Culturing Gastrointestinal Organoids,” Nature Communications 13 (2022): 1692. 10.1038/s41467-022-29279-4 PMC8967832 35354790

[115] D. M. Maahs , N. A. West , J. M. Lawrence , and E. J. Mayer‐Davis , “Chapter 1: Epidemiology of Type 1 Diabetes,” Endocrinology and Metabolism Clinincs of North America 39 (2010): 481–497. 10.1016/j.ecl.2010.05.011 PMC2925303 20723815

[116] E. A. C. de Waard , T. A. C. M. van Geel , H. H. C. M. Savelberg , A. Koster , P. P. M. M. Geusens , and J. P. W. van den Bergh , “Increased Fracture Risk in Patients with Type 2 Diabetes Mellitus: an Overview of the Underlying Mechanisms and the Usefulness of Imaging Modalities and Fracture Risk Assessment Tools,” Maturitas 79 (2014): 265–274. 25192916 10.1016/j.maturitas.2014.08.003

[117] D. R. Weber , K. Haynes , M. B. Leonard , S. M. Willi , and M. R. Denburg , “Type 1 Diabetes Is Associated With an Increased Risk of Fracture Across the Life Span: A Population‐Based Cohort Study Using The Health Improvement Network (THIN),” Diabetes Care 38 (2015): 1913–1920. 26216874 10.2337/dc15-0783 PMC4580610

[118] H. Wang , Y. Ba , Q. Xing , and J.‐L. Du , “Diabetes Mellitus and the Risk of Fractures at Specific Sites: A Meta‐Analysis,” BMJ Open 9 (2019): 024067. 10.1136/bmjopen-2018-024067 PMC6326306 30610024

[119] M. Janghorbani , R. M. Van Dam , W. C. Willett , and F. B. Hu , “Systematic Review of Type 1 and Type 2 Diabetes Mellitus and Risk of Fracture,” American Journal of Epidemiology 166 (2007): 495–505. 17575306 10.1093/aje/kwm106

[120] S. Khosla , P. Samakkarnthai , D. G. Monroe , and J. N. Farr , “Update on the Pathogenesis and Treatment of Skeletal Fragility in Type 2 Diabetes Mellitus,” Nature Reviews Endocrinology 17 (2021): 685–697. 10.1038/s41574-021-00555-5 PMC8605611 34518671

[121] A. V. Schwartz , “Association of BMD and FRAX Score with Risk of Fracture in Older Adults with Type 2 Diabetes,” Jama 305 (2011): 2184–2192. 21632482 10.1001/jama.2011.715 PMC3287389

[122] Q. Gao , Y. Jiang , D. Zhou , et al., “Advanced Glycation End Products Mediate Biomineralization Disorder in Diabetic Bone Disease,” Cell Reports Medicine 5 (2024): 101694. 39173634 10.1016/j.xcrm.2024.101694 PMC11524989

[123] M. R. Rubin and J. M. Patsch , “Assessment of Bone Turnover and Bone Quality in Type 2 Diabetic Bone Disease: Current Concepts and Future Directions,” Bone Research 4 (2016): 16001. 27019762 10.1038/boneres.2016.1 PMC4802604

[124] A. Wędrychowicz , K. Sztefko , and J. B. Starzyk , “Sclerostin and Its Significance for Children and Adolescents with Type 1 Diabetes Mellitus (T1D),” Bone 120 (2019): 387–392. 30120991 10.1016/j.bone.2018.08.007

[125] A. Piccoli , F. Cannata , R. Strollo , et al., “Sclerostin Regulation, Microarchitecture, and Advanced Glycation End‐Products in the Bone of Elderly Women With Type 2 Diabetes,” Journal of Bone and Mineral Research 35 (2020): 2415–2422. 32777114 10.1002/jbmr.4153 PMC8143610

[126] M. F. Faienza , A. Ventura , M. Delvecchio , et al., “High Sclerostin and Dickkopf‐1 (DKK‐1) Serum Levels in Children and Adolescents with Type 1 Diabetes Mellitus,” The Journal of Clinical Endocrinology & Metabolism 102 (2017): 1174–1181. 28388723 10.1210/jc.2016-2371

[127] A. Gaudio , F. Privitera , I. Pulvirenti , E. Canzonieri , R. Rapisarda , and C. E. Fiore , “The Relationship between Inhibitors of the Wnt Signalling Pathway (sclerostin and Dickkopf‐1) and Carotid Intima‐Media Thickness in Postmenopausal Women With Type 2 Diabetes Mellitus,” Diabetes and Vascular Disease Research 11 (2014): 48–52. 24227537 10.1177/1479164113510923

[128] Y. An , H. Zhang , C. Wang , et al., “Activation of ROS/MAPK s /NF‐ κ B/NLRP3 and Inhibition of Efferocytosis in Osteoclast‐Mediated Diabetic Osteoporosis,” The FASEB Journal 33 (2019): 12515–12527. 31461386 10.1096/fj.201802805RR PMC6902677

[129] D. M. Pacicca , T. Brown , D. Watkins , et al., “Elevated Glucose Acts Directly on Osteocytes to Increase Sclerostin Expression in Diabetes,” Scientific Reports 9 (2019): 17353. 31757981 10.1038/s41598-019-52224-3 PMC6874765

[130] X. Liu , W. Li , J. Cai , et al., “Spatiotemporal Characterization of Microdamage Accumulation and Its Targeted Remodeling Mechanisms in Diabetic Fatigued Bone,” The FASEB Journal 34 (2020): 2579–2594. 31908007 10.1096/fj.201902011RR

[131] K. Asadipooya and E. M. Uy , “Advanced Glycation End Products (AGEs), Receptor for AGEs, Diabetes, and Bone: Review of the Literature,” Journal of the Endocrine Society 3 (2019): 1799–1818. 31528827 10.1210/js.2019-00160 PMC6734192

[132] S. Y. Tang , U. Zeenath , and D. Vashishth , “Effects of Non‐enzymatic Glycation on Cancellous Bone Fragility,” Bone 40 (2007): 1144–1151. 17257914 10.1016/j.bone.2006.12.056 PMC4398019

[133] C. J. Thomas , T. P. Cleland , G. E. Sroga , and D. Vashishth , “Accumulation of Carboxymethyl‐Lysine (CML) in Human Cortical Bone,” Bone 110 (2018): 128–133. 29408699 10.1016/j.bone.2018.01.028 PMC5878737

[134] M. Saito , K. Fujii , Y. Mori , and K. Marumo , “Role of Collagen Enzymatic and Glycation Induced Cross‐links as a Determinant of Bone Quality in Spontaneously Diabetic WBN/Kob Rats,” Osteoporosis International 17 (2006): 1514–1523. 16770520 10.1007/s00198-006-0155-5

[135] A. D. McCarthy , S. B. Etcheverry , L. Bruzzone , G. Lettieri , D. A. Barrio , and A. M. Cortizo , “Non‐Enzymatic Glycosylation of a Type I Collagen Matrix: Effects on Osteoblastic Development and Oxidative Stress,” BMC Cell Biology 2 (2001): 16. 11518540 10.1186/1471-2121-2-16 PMC37548

[136] U. Valcourt , B. Merle , E. Gineyts , S. P. Viguet‐Carrin , P. D. Delmas , and P. Garnero , “Non‐Enzymatic Glycation of Bone Collagen Modifies Osteoclastic Activity and Differentiation,” Journal of Biological Chemistry 282 (2007): 5691–5703. 17142454 10.1074/jbc.M610536200

[137] Y.‐Z. Cheng , S.‐L. Yang , J.‐Y. Wang , et al., “Irbesartan Attenuates Advanced Glycation End Products‐mediated Damage in Diabetes‐Associated Osteoporosis through the AGEs/RAGE Pathway,” Life Sciences 205 (2018): 184–192. 29702126 10.1016/j.lfs.2018.04.042

[138] R. R. Kalyani , S. H. Golden , and W. T. Cefalu , “Diabetes and Aging: Unique Considerations and Goals of Care,” Diabetes Care 40 (2017): 440–443. 28325794 10.2337/dci17-0005 PMC5360288

[139] C. López‐Otín , M. A. Blasco , L. Partridge , M. Serrano , and G. Kroemer , “The Hallmarks of Aging,” Cell 153 (2013): 1194–1217. 23746838 10.1016/j.cell.2013.05.039 PMC3836174

[140] E. J. Moerman , K. Teng , D. A. Lipschitz , and B. Lecka‐Czernik , “Aging Activates Adipogenic and Suppresses Osteogenic Programs in Mesenchymal Marrow Stroma/Stem Cells: the Role of PPAR‐γ2 Transcription Factor and TGF‐β/BMP Signaling Pathways,” Aging Cell 3 (2004): 379–389. 15569355 10.1111/j.1474-9728.2004.00127.x PMC1850101

[141] S. Zhou , J. S. Greenberger , M. W. Epperly , et al., “Age‐Related Intrinsic Changes in human Bone‐Marrow‐Derived Mesenchymal Stem Cells and Their Differentiation to Osteoblasts,” Aging Cell 7 (2008): 335–343. 18248663 10.1111/j.1474-9726.2008.00377.x PMC2398731

[142] R. P. F. Abuna , C. T. Stringhetta‐Garcia , L. P. Fiori , R. C. M. Dornelles , A. L. Rosa , and M. M. Beloti , “Aging Impairs Osteoblast Differentiation of Mesenchymal Stem Cells Grown on Titanium by Favoring Adipogenesis,” Journal of Applied Oral Science 24 (2016): 376–382. 27556209 10.1590/1678-775720160037 PMC4990367

[143] A. Al Saedi , S. Bermeo , L. Plotkin , D. E. Myers , and G. Duque , “Mechanisms of Palmitate‐Induced Lipotoxicity in Osteocytes,” Bone 127 (2019): 353–359. 31226530 10.1016/j.bone.2019.06.016

[144] M. Almeida , E. Ambrogini , L. Han , S. C. Manolagas , and R. L. Jilka , “Increased Lipid Oxidation Causes Oxidative Stress, Increased Peroxisome Proliferator‐Activated Receptor‐γ Expression, and Diminished Pro‐Osteogenic Wnt Signaling in the Skeleton,” Journal of Biological Chemistry 284 (2009): 27438–27448. 19657144 10.1074/jbc.M109.023572 PMC2785673

[145] T. Su , M. Xu , F. Lu , and Q. Chang , “Adipogenesis or Osteogenesis: Destiny Decision Made by Mechanical Properties of Biomaterials,” RSC Advances 12 (2022): 24501–24510. 36128379 10.1039/d2ra02841g PMC9425444

[146] V. Sundararaghavan , M. M. Mazur , B. Evans , J. Liu , and N. A. Ebraheim , “Diabetes and Bone Health: Latest Evidence and Clinical Implications,” Therapeutic Advances in Musculoskeletal Disease 9 (2017): 67–74. 28344668 10.1177/1759720X16687480 PMC5349336

[147] A. Lin , F. Sved Skottvoll , S. Rayner , et al., “3D cell Culture Models and Organ‐on‐a‐Chip: Meet Separation Science and Mass Spectrometry,” Electrophoresis 41 (2020): 56–64. 31544246 10.1002/elps.201900170

[148] S. Chen , X. Chen , Z. Geng , and J. Su , “The Horizon of Bone Organoid: A Perspective on Construction and Application,” Bioactive Materials 18 (2022): 15–25. 35387160 10.1016/j.bioactmat.2022.01.048 PMC8961298

[149] R. Bortell and C. Yang , “The BB Rat as a Model of Human Type 1 Diabetes,” in Animal Models in Diabetes Research, eds. H.‐G. Joost , H. Al‐Hasani , and A. Schürmann , (Humana Press, 2012), 31–44, 10.1007/978-1-62703-068-7_3. 22893399

[150] J. H. Lee , S. H. Yang , J. M. Oh , and M. G. Lee , “Pharmacokinetics of Drugs in Rats with Diabetes Mellitus Induced by Alloxan or Streptozocin: Comparison With Those in Patients with Type I Diabetes Mellitus,” Journal of Pharmacy and Pharmacology 62 (2010): 1–23. 20722995 10.1211/jpp.62.01.0001

[151] A. J. F. King , “The Use of Animal Models in Diabetes Research,” British Journal of Pharmacology 166 (2012): 877–894. 22352879 10.1111/j.1476-5381.2012.01911.x PMC3417415

[152] T. Shi , K. Lu , S. Shen , et al., “Fenofibrate Decreases the Bone Quality by Down Regulating Runx2 in High‐Fat‐Diet Induced Type 2 Diabetes Mellitus Mouse Model,” Lipids in Health and Disease 16 (2017): 201. 29029615 10.1186/s12944-017-0592-5 PMC5640963

[153] R. Burcelin , V. Crivelli , A. Dacosta , A. Roy‐Tirelli , and B. Thorens , “Heterogeneous Metabolic Adaptation of C57BL/6J Mice to High‐Fat Diet,” American Journal of Physiology‐Endocrinology and Metabolism 282 (2002): E834–E842. 11882503 10.1152/ajpendo.00332.2001

[154] D. N. Athmuri and P. A. Shiekh , “Experimental Diabetic Animal Models to Study Diabetes and Diabetic Complications,” MethodsX 11 (2023): 102474. 38023309 10.1016/j.mex.2023.102474 PMC10661736

[155] N. Y. Y. Koh , J. J. Miszkiewicz , M. L. Fac , N. K. Y. Wee , and N. A. Sims , “Preclinical Rodent Models for Human Bone Disease, Including a Focus on Cortical Bone,” Endocrine Reviews 45 (2024): 493–520, 10.1210/endrev/bnae004. 38315213 PMC11244217

[156] T. Freude , K. F. Braun , A. Haug , et al., “Hyperinsulinemia Reduces Osteoblast Activity In Vitro via Upregulation of TGF‐β,” Journal of Molecular Medicine 90 (2012): 1257–1266. 22926010 10.1007/s00109-012-0948-2

[157] M. Susa , N.‐H. Luong‐Nguyen , D. Cappellen , N. Zamurovic , and R. Gamse , “Human Primary Osteoclasts: In Vitro Generation and Applications as Pharmacological and Clinical Assay,” Journal of Translational Medicine 2 (2004): 6. 15025786 10.1186/1479-5876-2-6 PMC394349

[158] S. Fuchs , A. Hofmann , and C. J. Kirkpatrick , “Microvessel‐Like Structures from Outgrowth Endothelial Cells from Human Peripheral Blood in 2‐Dimensional and 3‐Dimensional Co‐Cultures with Osteoblastic Lineage Cells,” Tissue Engineering 13 (2007): 2577–2588. 17655487 10.1089/ten.2007.0022

[159] K. Keklikoglou , C. Arvanitidis , G. Chatzigeorgiou , et al., “Micro‐CT for Biological and Biomedical Studies: a Comparison of Imaging Techniques,” Journal of Imaging 7 (2021): 172. 34564098 10.3390/jimaging7090172 PMC8470083

[160] K. Hygum , J. Starup‐Linde , T. Harsløf , P. Vestergaard , and B. L. Langdahl , “Mechanisms In Endocrinology: Diabetes Mellitus, a State of Low Bone Turnover—A Systematic Review and Meta‐analysis,” European Journal of Endocrinology 176 (2017): R137–R157. 28049653 10.1530/EJE-16-0652

[161] I. Miguel‐Escalada , S. Bonàs‐Guarch , I. Cebola , et al., “Human Pancreatic Islet Three‐dimensional Chromatin Architecture Provides Insights into the Genetics of Type 2 Diabetes,” Nature Genetics 51 (2019): 1137–1148. 31253982 10.1038/s41588-019-0457-0 PMC6640048

[162] P. Mali , L. Yang , K. M. Esvelt , et al., “RNA‐Guided Human Genome Engineering via Cas9,” Science 339 (2013): 823–826. 23287722 10.1126/science.1232033 PMC3712628

[163] Z. Zhu , Q. V. Li , K. Lee , et al., “Genome Editing of Lineage Determinants in Human Pluripotent Stem Cells Reveals Mechanisms of Pancreatic Development and Diabetes,” Cell Stem Cell 18 (2016): 755–768. 27133796 10.1016/j.stem.2016.03.015 PMC4892994

[164] C. Medeiros and J. M. Wallace , “High Glucose‐Induced Inhibition of Osteoblast like MC3T3‐E1 Differentiation Promotes Mitochondrial Perturbations,” PLoS ONE 17 (2022): 0270001. 10.1371/journal.pone.0270001 PMC9205493 35714142

[165] A. Dienelt and N. I. zur Nieden , “Hyperglycemia Impairs Skeletogenesis From Embryonic Stem Cells by Affecting Osteoblast and Osteoclast Differentiation,” Stem Cells and Development 20 (2011): 465–474. 20939707 10.1089/scd.2010.0205

[166] K.‐C. Huang , P.‐Y. Chuang , T.‐Y. Yang , T.‐W. Huang , and S.‐F. Chang , “Hyperglycemia Inhibits Osteoblastogenesis of Rat Bone Marrow Stromal Cells via Activation of the Notch2 Signaling Pathway,” International Journal of Medical Sciences 16 (2019): 696–703. 31217737 10.7150/ijms.32707 PMC6566748

[167] A. Takeno , I. Kanazawa , K.‐I. Tanaka , et al., “High Glucose Promotes Mineralization via Bone Morphogenetic Protein 4‐Smad Signals in Early Stage of Osteoblast Differentiation,” Diabetology International 12 (2021): 171–180. 33786272 10.1007/s13340-020-00463-5 PMC7943678

[168] L. de Lemos , P. Antas , I. S. Ferreira , et al., “Modelling Neurodegeneration and Inflammation in Early Diabetic Retinopathy Using 3D Human Retinal Organoids,” In Vitro models 3 (2024): 33–48. 39872068 10.1007/s44164-024-00068-1 PMC11756505

[169] H. Naderi‐Meshkin , M. Eleftheriadou , G. Carney , et al., “BS16 Blood Vessel Organoids Derived from Diabetic Patients Revealed Impaired Function Based on a Subpopulation of Endothelial Cells,” Heart 109 (2023): A256–A257.

[170] N. Jalava , M. Arponen , N. Widjaja , T. J. Heino , and K. K. Ivaska , “Short‐ and Long‐term Exposure to High Glucose Induces Unique Transcriptional Changes in Osteoblasts In Vitro,” Biology Open 13 (2024): bio060239. 38742438 10.1242/bio.060239 PMC11128269

[171] V. P. Singh , A. Bali , N. Singh , and A. S. Jaggi , “Advanced Glycation End Products and Diabetic Complications. The Korean Journal of Physiology & Pharmacology ,” Official Journal of the Korean Physiological Society and the Korean Society of Pharmacology 18 (2014): 1. 10.4196/kjpp.2014.18.1.1 PMC3951818 24634591

[172] F. A. C. Jansen , J. Rubert , K. van Norren , V. Fogliano , and T. Hoppenbrouwers , “Seeking Standardized In Vitro Models of AGE‐RAGE Signaling in the Physiological Perspective of Glycated Dietary Proteins,” International Journal of Biological Macromolecules 318 (2025): 144889. 40466838 10.1016/j.ijbiomac.2025.144889

[173] G. E. Sroga and D. Vashishth , “Controlled Formation of Carboxymethyllysine in Bone Matrix Through Designed Glycation Reaction,” JBMR Plus 5 (2021): 10548. 10.1002/jbm4.10548 PMC8567485 34761150

[174] K. Fulzele , R. C. Riddle , D. J. DiGirolamo , et al., “Insulin Receptor Signaling in Osteoblasts Regulates Postnatal Bone Acquisition and Body Composition,” Cell 185 (2022): 746. 35180388 10.1016/j.cell.2022.01.016 PMC8959017

[175] M. Ferron , J. Wei , T. Yoshizawa , et al., “Insulin Signaling in Osteoblasts Integrates Bone Remodeling and Energy Metabolism,” Cell 142 (2010): 296–308. 20655470 10.1016/j.cell.2010.06.003 PMC2910411

[176] M. Pi , Y. Wu , and L. D. Quarles , “GPRC6A mediates Responses to Osteocalcin in β‐Cells In Vitro and Pancreas In Vivo,” Journal of Bone and Mineral Research 26 (2011): 1680–1683. 21425331 10.1002/jbmr.390 PMC5079536

[177] A. Mizokami , Y. Yasutake , J. Gao , et al., “Osteocalcin Induces Release of Glucagon‐Like Peptide‐1 and Thereby Stimulates Insulin Secretion in Mice,” PLoS ONE 8 (2013): 57375. 10.1371/journal.pone.0057375 PMC3577726 23437377

[178] Y. Hu , H. Zhang , S. Wang , et al., “Bone/Cartilage Organoid on‐chip: Construction Strategy and Application,” Bioactive Materials 25 (2023): 29–41. 37056252 10.1016/j.bioactmat.2023.01.016 PMC10087111

[179] Z. Li , Z. Lin , S. Liu , et al., “Human Mesenchymal Stem Cell‐Derived Miniature Joint System for Disease Modeling and Drug Testing,” Advanced Science 9 (2022): 2105909. 35436042 10.1002/advs.202105909 PMC9313499

[180] E. L. George , S. L. Truesdell , S. L. York , and M. M. Saunders , “Lab‐on‐a‐Chip Platforms for Quantification of Multicellular Interactions in Bone Remodeling,” Experimental Cell Research 365 (2018): 106–118. 29499205 10.1016/j.yexcr.2018.02.027

[181] K. Kozuka , Y. He , S. Koo‐McCoy , et al., “Development and Characterization of a Human and Mouse Intestinal Epithelial Cell Monolayer Platform,” Stem Cell Reports 9 (2017): 1976–1990. 29153987 10.1016/j.stemcr.2017.10.013 PMC5785676

[182] L. Broutier , G. Mastrogiovanni , M. M. Verstegen , et al., “Human Primary Liver Cancer–derived Organoid Cultures for Disease Modeling and Drug Screening,” Nature Medicine 23 (2017): 1424–1435. 10.1038/nm.4438 PMC5722201 29131160

[183] T. C. Brennan‐Speranza , H. Henneicke , S. J. Gasparini , et al., “Osteoblasts Mediate the Adverse Effects of Glucocorticoids on Fuel Metabolism,” Journal of Clinical Investigation 122 (2012): 4172–4189. 23093779 10.1172/JCI63377 PMC3484445

[184] X. Chen , N. Lu , S. Huang , Y. Zhang , Z. Liu , and X. Wang , “Assessment of Doxorubicin Toxicity Using human Cardiac Organoids: a Novel Model for Evaluating Drug Cardiotoxicity,” Chemico‐Biological Interactions 386 (2023): 110777. 37879593 10.1016/j.cbi.2023.110777

[185] I. R. König , O. Fuchs , G. Hansen , E. V. Mutius , and M. V. Kopp , “What Is Precision Medicine?,” European Respiratory Journal 50 (2017): 1700391. 29051268 10.1183/13993003.00391-2017

[186] C. J. de Witte , J. Espejo Valle‐Inclan , N. Hami , et al., “Patient‐Derived Ovarian Cancer Organoids Mimic Clinical Response and Exhibit Heterogeneous Inter‐ and Intrapatient Drug Responses,” Cell Reports 31 (2020): 107762. 32553164 10.1016/j.celrep.2020.107762

[187] O. Kopper , C. J. de Witte , K. Lõhmussaar , et al., “An Organoid Platform for Ovarian Cancer Captures Intra‐ and Interpatient Heterogeneity,” Nature Medicine 25 (2019): 838–849. 10.1038/s41591-019-0422-6 31011202

[188] G. Vlachogiannis , S. Hedayat , A. Vatsiou , et al., “Patient‐derived Organoids Model Treatment Response of Metastatic Gastrointestinal Cancers,” Science 359 (2018): 920–926. 29472484 10.1126/science.aao2774 PMC6112415

[189] S. N. Ooft , F. Weeber , K. K. Dijkstra , et al., “Patient‐Derived Organoids Can Predict Response to Chemotherapy in Metastatic Colorectal Cancer Patients,” Science Translational Medicine 11 (2019): aay2574. 10.1126/scitranslmed.aay2574 31597751

[190] H.‐Y. Kim , C. Charton , J. H. Shim , et al., “Patient‐Derived Organoids Recapitulate Pathological Intrinsic and Phenotypic Features of Fibrous Dysplasia,” Cells 13 (2024): 729. 38727265 10.3390/cells13090729 PMC11083396

[191] C. Wang , W. Huang , Y. Zhou , et al., “3D printing of Bone Tissue Engineering Scaffolds,” Bioactive Materials 5 (2020): 82–91. 31956737 10.1016/j.bioactmat.2020.01.004 PMC6962643

[192] K. Mei , P. Pasyar , M. Geagan , et al., “Design and Fabrication of 3D‐Printed Patient‐Specific Soft Tissue and Bone Phantoms for CT Imaging,” Scientific Reports 13 (2023): 17495. 37840044 10.1038/s41598-023-44602-9 PMC10577126

[193] Q. Yang , M. Li , Z. Xiao , Y. Feng , L. Lei , and S. Li , “A New Perspective on Precision Medicine: The Power of Digital Organoids,” Biomaterials Research 29 (2025): 0171. 40129676 10.34133/bmr.0171 PMC11931648

[194] E. Driehuis and H. Clevers , “CRISPR/Cas 9 Genome Editing and Its Applications in Organoids,” American Journal of Physiology‐Gastrointestinal and Liver Physiology 312 (2017): G257–G265. 28126704 10.1152/ajpgi.00410.2016

[195] S. Huang , Y. Wu , H. Zhao , et al., “Advancements in Bone Organoids: Perspectives on Construction Methodologies and Application Strategies,” Journal of Advanced Research 81 (2026): 745–767. 40513657 10.1016/j.jare.2025.06.011 PMC12957805

[196] B. Bouvard and G. Mabilleau , “Gut Hormones and Bone Homeostasis: Potential Therapeutic Implications,” Nature Reviews Endocrinology 20 (2024): 553–564. 10.1038/s41574-024-01000-z 38858581

[197] P. Upadhyay and S. Kumar , “Diabetes and Bone Health: A Comprehensive Review of Impacts and Mechanisms,” Diabetes/Metabolism Research and Reviews 41 (2025): 70062. 10.1002/dmrr.70062 40557919

[198] K.‐C. Huang , C.‐Y. Lin , P.‐Y. Chuang , et al., “Microbiota Diversity and Its Influence on Diabetic Osteoporosis Development,” Biochemical and Biophysical Research Communications 790 (2025): 152884. 41172807 10.1016/j.bbrc.2025.152884

[199] V. Bozzetti and S. Senger , “Organoid Technologies for the Study of Intestinal Microbiota–host Interactions,” Trends in Molecular Medicine 28 (2022): 290–303. 35232671 10.1016/j.molmed.2022.02.001 PMC8957533

[200] N. Li , X. Dai , F. Yang , et al., “Spontaneous Spheroids from Alveolar Bone‐derived Mesenchymal Stromal Cells Maintain Pluripotency of Stem Cells by Regulating Hypoxia‐inducible Factors,” Biological Research 56 (2023): 17. 37016436 10.1186/s40659-023-00421-w PMC10074860

[201] T. Zhang , S. Lin , X. Shao , et al., “Effect of Matrix Stiffness on Osteoblast Functionalization,” Cell Proliferation 50 (2017): 12338. 10.1111/cpr.12338 PMC6529113 28205330

[202] C. A. Mullen , M. G. Haugh , M. B. Schaffler , R. J. Majeska , and L. M. McNamara , “Osteocyte Differentiation Is Regulated by Extracellular Matrix Stiffness and Intercellular Separation,” Journal of the Mechanical Behavior of Biomedical Materials 28 (2013): 183–194. 23994943 10.1016/j.jmbbm.2013.06.013 PMC5776008

[203] Z. Gan , X. Qin , H. Liu , J. Liu , and J. Qin , “Recent Advances in Defined Hydrogels in Organoid Research,” Bioactive Materials 28 (2023): 386–401. 37334069 10.1016/j.bioactmat.2023.06.004 PMC10273284

[204] R. E. Unger , S. Ghanaati , C. Orth , et al., “The Rapid Anastomosis between Prevascularized Networks on Silk Fibroin Scaffolds Generated In Vitro with Cocultures of human Microvascular Endothelial and Osteoblast Cells and the Host Vasculature,” Biomaterials 31 (2010): 6959–6967. 20619788 10.1016/j.biomaterials.2010.05.057

[205] X. Sun , X. Jiao , X. Yang , et al., “3D bioprinting of Osteon‐mimetic Scaffolds With Hierarchical Microchannels for Vascularized Bone Tissue Regeneration,” Biofabrication 14 (2022): 035008. 10.1088/1758-5090/ac6700 35417902

[206] Y. Zhang , T. Sun , J. Cheng , et al., “Osteon‐Inspired Dual‐Ring Hydrogel Scaffold with Spatially Programmed Cell Encapsulation for Enhanced Angiogenesis and Osteogenesis in Bone Repair,” ACS Applied Materials & Interfaces 17 (2025): 42849–42862. 40668406 10.1021/acsami.5c10673

[207] L. Novikova , I. V. Smirnova , S. Rawal , A. L. Dotson , S. H. Benedict , and L. Stehno‐Bittel , “Variations in Rodent Models of Type 1 Diabetes: Islet Morphology,” Journal of Diabetes Research 2013 (2013): 1. 10.1155/2013/965832 PMC3671304 23762878

[208] J.‐Y. Fang , C.‐H. Lin , T.‐H. Huang , and S.‐Y. I. Chuang , “Vivo Rodent Models of Type 2 Diabetes and Their Usefulness for Evaluating Flavonoid Bioactivity,” Nutrients 11 (2019): 530. 30823474 10.3390/nu11030530 PMC6470730

[209] S. Marino , N. Akel , S. Li , et al., “Reversal of the Diabetic Bone Signature With Anabolic Therapies in Mice,” Bone Research 11 (2023): 19. 37076478 10.1038/s41413-023-00261-0 PMC10115794

[210] V. Uribe and A. Rosello‐Diez , “Culturing and Measuring Fetal and Newborn Murine Long Bones,” JoVE (Journal of Visualized Experiments) (2019): 59509, 10.3791/59509. 31081827

[211] I. Yuste , F. C. Luciano , E. González‐Burgos , A. Lalatsa , and D. R. Serrano , “Mimicking Bone Microenvironment: 2D and 3D In Vitro Models of Human Osteoblasts,” Pharmacological Research 169 (2021): 105626. 33892092 10.1016/j.phrs.2021.105626

